# IGF2BP2 regulates the inflammation of fibroblast-like synoviocytes via GSTM5 in rheumatoid arthritis

**DOI:** 10.1038/s41420-024-01988-3

**Published:** 2024-05-03

**Authors:** Yunyi Nan, Minhao Chen, Weijie Wu, Rongrong Huang, Weiwei Sun, Qian Lu, Zhifeng Gu, Xingxing Mao, Hua Xu, Youhua Wang

**Affiliations:** 1 Department of Orthopaedics, Affiliated Hospital of Nantong University, Medical School of Nantong University, 226001 Nantong, China; 2 Department of Orthopaedics, Affiliated Nantong Hospital of Shanghai University, The Sixth People’s Hospital of Nantong, 226001 Nantong, China; 3 Department of Pharmacy, Affiliated Hospital of Nantong University, 226001 Nantong, China; 4 Department of Rheumatology, Affiliated Hospital of Nantong University, 226001 Nantong, China

**Keywords:** Immunology, Autoimmunity

## Abstract

Rheumatoid arthritis (RA) is a chronic autoimmune disease with an unknown etiology. RA cannot be fully cured and requires lengthy treatment, imposing a significant burden on both individuals and society. Due to the lack of specific drugs available for treating RA, exploring a key new therapeutic target for RA is currently an important task. Activated fibroblast-like synoviocytes (FLSs) play a crucial role in the progression of RA, which release interleukin (IL)-1β, IL-6 and tumor necrosis factor (TNF)-α resulting in abnormal inflammatory reaction in the synovium. A previous study has highlighted the correlation of m^6^A reader insulin-like growth factor 2 mRNA-binding protein 2 (IGF2BP2) with inflammation-related diseases in human. However, the role of IGF2BP2 in the inflammatory reaction of FLSs during RA progression has not been assessed. In this study, IGF2BP2 expression was decreased in the synovial tissues of RA patients and collagen-induced arthritis (CIA) rats. Intra-articular injection of an adeno-associated virus (AAV) vector overexpressing *IGF2BP2* relieved paw swelling, synovial hyperplasia and cartilage destruction in CIA rats. IGF2BP2 overexpression also inhibited lipopolysaccharide (LPS)-mediated RA fibroblast-like synoviocytes (RA-FLSs) migration and invasion accompanied by a decreased level of inflammatory factors in vitro. Conversely, IGF2BP2 suppression promoted RA-FLSs migration and invasion with an elevated level of inflammatory factors in vitro. The sequencing result showed that glutathione S-transferase Mu 5 (*GSTM5*), a key antioxidant gene, was the target mRNA of *IGF2BP2*. Further experiments demonstrated that IGF2BP2 strengthened the stability of *GSTM5* mRNA, leading to weakened inflammatory reaction and reduced expression of matrix metalloproteinase 9 and 13 (MMP9, MMP13). Therefore, IGF2BP2-GSTM5 axis may represent a potential therapeutic target for RA treatment.

## Introduction

Rheumatoid arthritis (RA) is an autoimmune disease that requires long-term treatment and imposes a heavy burden on both individuals and society. Epidemiological studies have shown that the incidence of RA is 0.5–1%, and it can not be completely cured at present [1]. Currently, the primary treatment options for RA include surgery and medication, such as disease-modifying antirheumatic drugs, non-steroidal anti-inflammatory drugs, glucocorticoids, biologics, and small molecule targeted drugs. Although these treatments may alleviate the synovitis and systemic inflammation, the adverse reactions of these drugs to various organs cannot be ignored. Moreover, the absence of targeted pharmaceutical interventions for the treatment of RA poses a significant challenge, primarily due to the presence of diverse comorbidities in RA patients, such as coronary heart disease, hypertension, interstitial lung disease, malignant tumors, osteoporosis, and the complex and varied clinical manifestations associated with the disease [2, 3]. Therefore, the management of RA patients continues to encounter numerous obstacles and difficulties. Notably, activated fibroblast-like synoviocytes (FLSs), which exhibit similar characteristics to tumor cells, assume a pivotal role in the progression of RA. As the disease progresses, the synovium undergoes a transformation into proliferative and invasive tissue, leading to cartilage and bone damage [4]. The excessive FLSs release interleukin (IL)-1β, IL-6 and tumor necrosis factor (TNF)-α that cause abnormal inflammatory reaction in the synovium. Additionally, activated FLSs release matrix metalloproteinases (MMPs) that primarily contribute to the destruction of cartilage structure by decomposing chondrocytes and extracellular matrix [5]. However, the regulatory mechanism of MMPs in RA remains unclear.

N^6^-methyladenosine (m^6^A) is known as the methylation at the N6 position of adenosine in transcripts [6]. Cumulative evidence suggests that m^6^A is the most common, abundant and conserved internal transcriptional modification on eukaryotic messenger RNAs (mRNAs) [7]. RNA m^6^A methylation significantly influences RNA metabolism and is involved in almost all stages of the RNA cycle, including mRNA splicing, nuclear export, translation, stability and degradation [8]. Numerous studies have demonstrated that this modification is associated with the occurrence and development of multiple diseases, particularly in tumors and autoimmune diseases [9]. However, the connection between m^6^A methylation and RA remains unclear and requires further exploration [10]. The m^6^A modification is a dynamic and reversible process regulated by methyltransferases, demethylases and reader proteins [11]. Insulin-like growth factor 2 mRNA-binding protein 2 (IGF2BP2), a member of m^6^A read proteins, has been reported to enhance the stability of m^6^A-modified mRNAs and facilitate their translation [12]. In recent years, growing studies have shown that IGF2BP2 plays a critical role in regulating inflammation-related diseases. In autoantibody-induced glomerulonephritis, knockout of IGF2BP2 could decrease the expression of Lcn2 and ameliorate renal inflammation [13]. In experimental autoimmune encephalomyelitis, IGF2BP2 was located at the junction of a stromal cell/T cell autoimmune circuit that drove neuroinflammation through a CCL2-Th17 cell axis [14]. Meanwhile, IGF2BP2-deleted macrophages exhibited an enhanced M1 phenotype and promoted the development of dextran sulfate sodium-induced experimental colitis [15]. However, the functional role of IGF2BP2 in the occurrence and development of RA remains unclear.

In this study, we observed low expression of IGF2BP2 in the synovial tissues of both RA patients and collagen-induced arthritis (CIA) rats. The in-situ injection of an adeno-associated virus (AAV) vector carrying *IGF2BP2* reduced paw thickness and arthritis scores in CIA rats, accompanied by the alleviation of bone and cartilage erosion. Subsequently, we screened and confirmed that glutathione S-transferase Mu 5 (*GSTM5*) is a downstream gene of IGF2BP2, which has been reported as an antioxidant gene [16]. Further experiments indicated that IGF2BP2 directly binds to the coding sequence (CDS) region of *GSTM5* mRNA, stabilizing its mRNA in an m^6^A modification-dependent manner. This stabilization finally affects inflammatory response and the expression of MMP9 and MMP13. Overall, our study suggests that IGF2BP2 may serve as a potential therapeutic target for the treatment of RA.

## Results

### Decreased expression of IGF2BP2 in the synovial tissues of RA patients and CIA rats

**Fig. 1:**
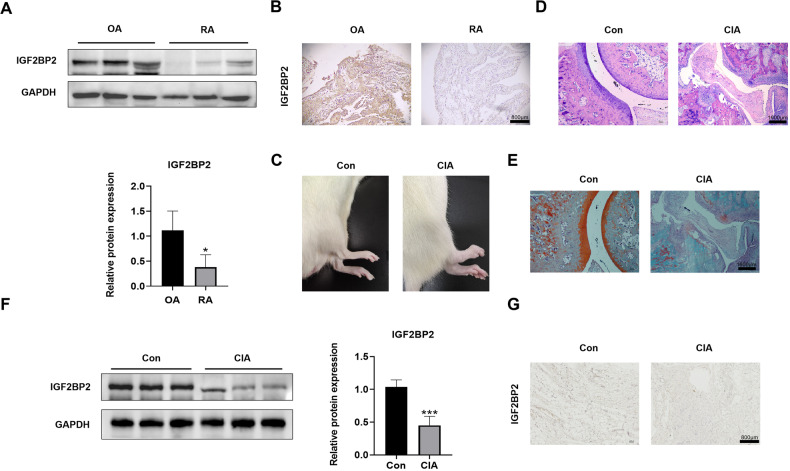
Decreased expression of IGF2BP2 in the synovial tissues of RA patients and CIA rats. **A** Western blotting was used to detect IGF2BP2 protein level in synovial tissues of OA and RA patients. **B** IHC staining of IGF2BP2 expression in synovial tissue specimens of OA and RA patients. Scale Bar = 800 µm. **C** Representative photographs of morphology in the normal group and the CIA group. **D**, **E** H&E and Safranin-O staining of joints in the normal group and CIA group. Bar = 1600 µm. **F** Western blotting was used to detect IGF2BP2 protein levels in synovial tissues of the normal group and CIA group (*n* = 6 per group). **G** IHC staining of IGF2BP2 expression in synovial tissue specimens of the normal group and CIA group. Scale Bar = 800 µm. ^*^P < 0.05, ^***^P < 0.001.

We initially assessed the expression levels of IGF2BP2 in the synovial tissues of patients with OA and RA by western blotting and IHC staining. In comparison to OA patients, the expression of IGF2BP2 was significantly reduced in RA patients (Fig. 1A, B and Fig. S1A). A representative photograph illustrated that there was marked redness and swelling in the paws of CIA rats (Fig. 1C). We then employed H&E and Safranin-O staining to examine ankle pathology changes. Compared to the control group, the CIA group exhibited significant aggravation in synovial hyperplasia, cartilage destruction and bone destruction (Fig. 1D, E). Additionally, representative blots showed a decrease in IGF2BP2 expression in the synovial tissues of CIA rats (Fig. 1F and Fig. S1B), which was further confirmed by IHC assay (Fig. 1G). Immunofluorescence staining revealed that there was strong co-localization of IGF2BP2 and Vimentin, a FLSs marker, in rat synovial tissues (Fig. S2). These findings suggested a reduction in IGF2BP2 expression in the synovial tissues of RA patients and CIA rats.

### Overexpression of IGF2BP2 alleviated the severity of arthritis in CIA rats

**Fig. 2:**
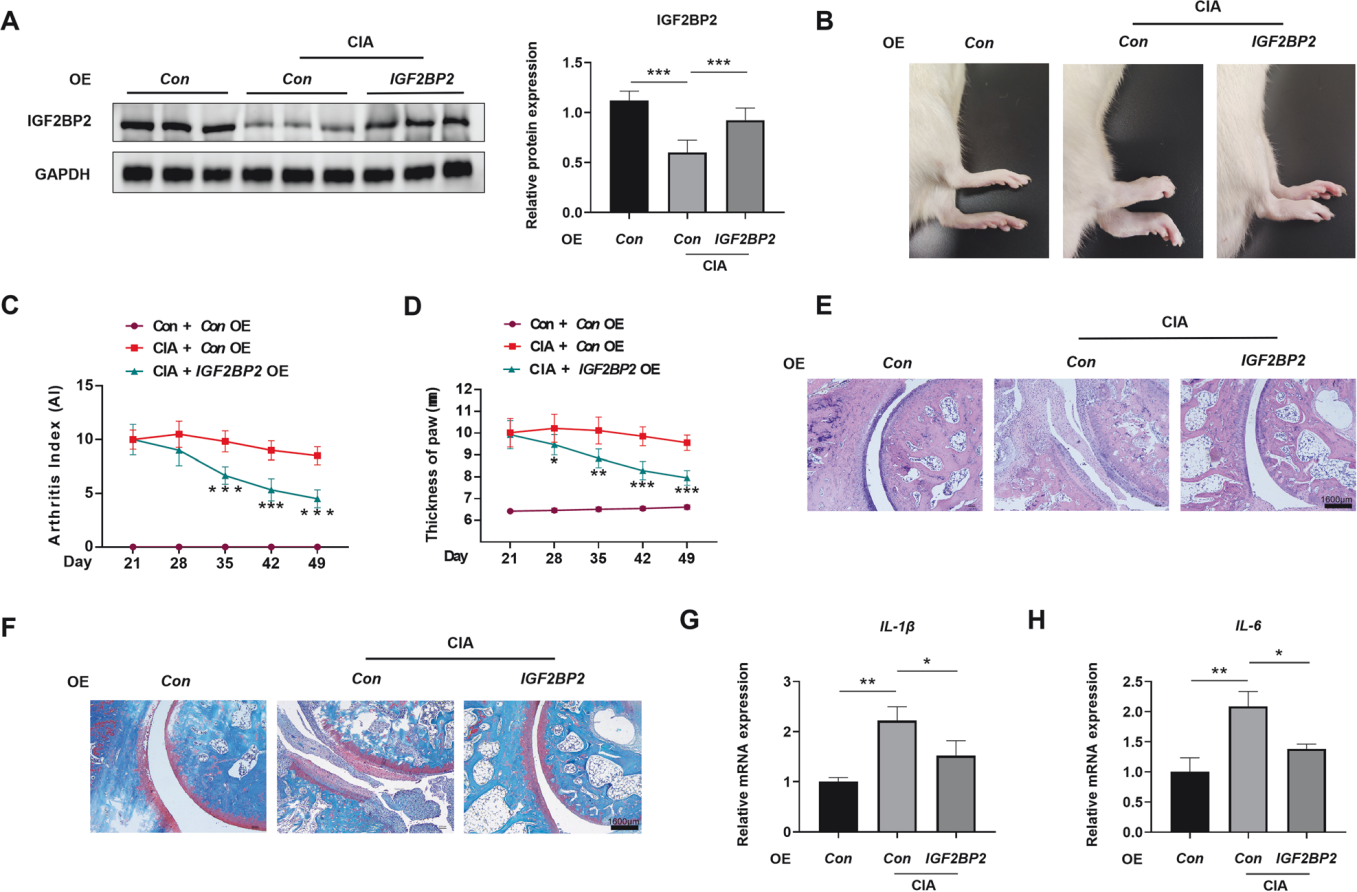
Overexpression of IGF2BP2 alleviated the severity of arthritis in CIA rats. **A** The expression of IGF2BP2 was detected by western blotting in synovial tissues of rats after *IGF2BP2* AAV treatment (*n* = 6 per group). **B** Representative photographs of morphology in different treatment groups. The arthritis index (**C**) and paw thickness (**D**) were scored and recorded every 7 days in a blinded manner. ^*^Compared with CIA. (**E**, **F**) H&E and Safranin-O staining of the ankle in different groups. Scale Bar = 1600 µm. **G**, **H** qPCR analysis of the mRNA expression of *IL-1β* and *IL-6* in different treatment groups. ^*^P < 0.05, ^**^P < 0.01, ^***^P < 0.001.

Subsequently, we employed the CIA model to examine the impact of IGF2BP2 on the progression of RA. Following booster immunization, CIA rats received an intra-articular injection of an AAV vector carrying *IGF2BP2* (Fig. S3A). The *IGF2BP2* overexpressive AAV significantly reversed the decrease in IGF2BP2 protein expression in the synovial tissues of CIA rats (Fig. 2A and Fig. S4), as well as *IGF2BP2* mRNA expression (Fig. S3B). In comparison to the CIA group, paw thickness and arthritis scores were diminished in CIA rats treated with *IGF2BP2* AAV (Fig. 2B–D). Meanwhile, histological assessments through H&E and Safranin-O staining revealed reduced synovial hyperplasia, cartilage degradation and bone destruction in *IGF2BP2*-treated CIA rats (Fig. 2E, F). Furthermore, qPCR results demonstrated that the expression levels of *IL-1β* and *IL-6* in the *IGF2BP2*-treated group were significantly lower than those in the CIA group (Fig. 2G, H). These results indicated that IGF2BP2 overexpression effectively attenuated the clinical symptoms of RA in CIA rats.

### IGF2BP2 positively regulated GSTM5 expression in the synovial tissues of CIA rats

**Fig. 3:**
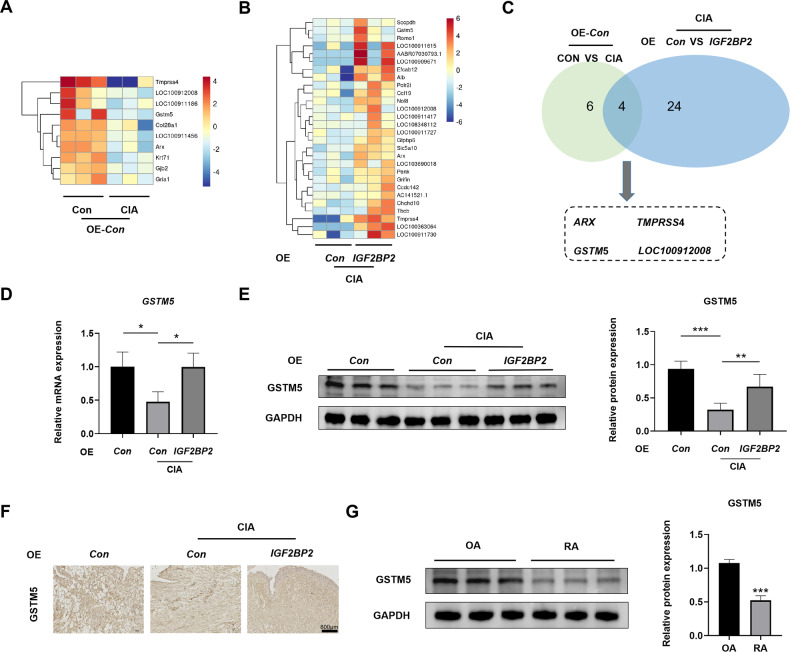
IGF2BP2 positively regulated GSTM5 expression in the synovial tissues of CIA rats. **A** Heatmap plot of DEGs between CON and CIA groups. **B** Heatmap plot of DEGs between CIA and AAV groups. **C** Venn diagram among 10 down-regulated genes and 28 up-regulated genes. **D** The mRNA expression of *GSTM5* in synovial tissues of rats was detected by qPCR after *IGF2BP2* AAV treatment. **E** The expression of GSTM5 was detected by western blotting in synovial tissues of rats after *IGF2BP2* AAV treatment (*n* = 6 per group). **F** IHC staining of GSTM5 expression in synovial tissue specimens of different groups. Scale Bar = 800 µm. **G** Western blotting was used to detect GSTM5 protein level in synovial tissues of OA and RA patients. ^*^P < 0.05, ^**^P < 0.01, ^***^P < 0.001.

To explore the downstream mechanism of IGF2BP2, transcriptome sequencing was conducted on the synovial tissues from CIA rats injected with control and *IGF2BP2* overexpressing AAV. As shown in Fig. 3A, 10 mRNA transcripts (|log_2_ Fold change| > 2, *P* < 0.05) were downregulated in the CIA group. As shown in Fig. 3B, 28 mRNA transcripts (|log_2_ Fold change| >2, *P* < 0.05) were upregulated in the CIA + *IGF2BP2* AAV group. Four mRNA transcripts exhibited consistent changes among the control, CIA and CIA + *IGF2BP2* AAV groups (Fig. 3C). These four mRNA transcripts were further validated in the synovial tissues of CIA rats. *GSTM5* mRNA was notably decreased in the CIA group, and this reduction was rescued by IGF2BP2 overexpression (Fig. 3D). The mRNA levels of *ARX* and *LOC100912008* showed similar changes but without significant statistical differences (Fig. S3C, D). The low abundance of *TMPRSS4* mRNA led to its being undetected in some rat synovial tissue samples (Table S2). Consequently, GSTM5 was the main focus of our study due to its potential role in RA. Representative blots demonstrated that GSTM5 expression was decreased in the CIA group, and IGF2BP2 overexpression restored GSTM5 levels (Fig. 3E and Fig. S5A). This finding was further confirmed by IHC assays (Fig. 3F). Moreover, GSTM5 expression was significantly decreased in RA patients compared to OA patients (Fig. 3G and Fig. S5B). Immunofluorescence staining in rat synovial tissues revealed the co-localization of GSTM5 and Vimentin. Furthermore, the expression of GSTM5 was downregulated consistently with IGF2BP2, as mentioned above (Fig. S6). These results suggested that IGF2BP2 promoted GSTM5 expression in RA.

### IGF2BP2 inhibited the migration, invasion, and inflammatory reaction of RA-FLSs

**Fig. 4:**
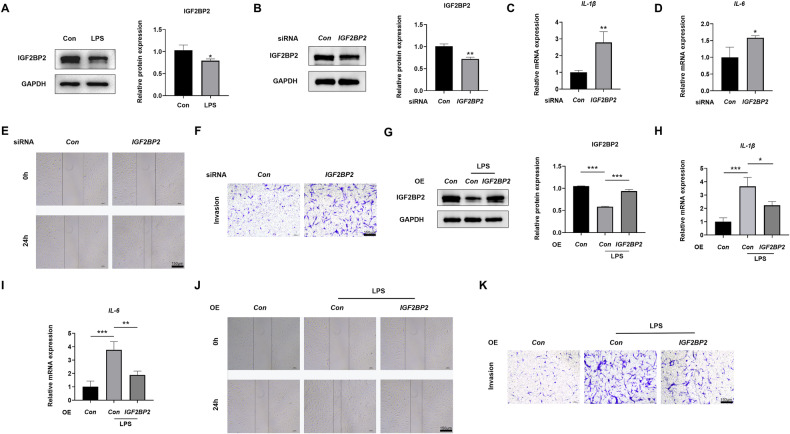
IGF2BP2 inhibited the migration, invasion, and inflammatory reactions of RA-FLSs. **A** Western blotting was used to detect IGF2BP2 protein level in RA-FLSs after treatment with LPS. **B** Western blotting was used to detect IGF2BP2 protein level in RA-FLSs after treatment with *IGF2BP2* siRNA. **C**, **D** qPCR analysis of the mRNA expression of *IL-1β* and *IL-6* in RA-FLSs after treatment with *IGF2BP2* siRNA. **E**, **F** Wound healing and transwell invasion assays showed the capacity of migration and invasion of RA-FLSs after treatment with *IGF2BP2* siRNA. Scale Bar = 150 µm. **G** IGF2BP2 protein level in LPS-incubated RA-FLSs after overexpressing *IGF2BP2*, as detected by western blotting. **H**, **I** qPCR analysis of the mRNA expression of *IL-1β* and *IL-6* in LPS-incubated RA-FLSs after treatment with *IGF2BP2* overexpression lentivirus. **J**, **K** Wound healing and transwell invasion assays showed the capacity of migration and invasion of LPS-incubated RA-FLSs after overexpressing *IGF2BP2*. Scale Bar = 150 µm. ^*^P < 0.05, ^**^P < 0.01, ^***^P < 0.001.

Having determined the effect of IGF2BP2 on CIA rats, our subsequent focus was to investigate its role in FLSs derived from RA synovial tissues (Fig. S7A). Exposure to LPS resulted in reduced expression of IGF2BP2 in RA-FLSs (Fig. 4A and Fig. S8A). SiRNAs targeting *IGF2BP2* mRNA were designed to downregulate IGF2BP2, and *IGF2BP2* siRNA1 showed the most effective knockdown efficiency at the protein level (Fig. 4B, Fig. S7B, Fig. S8B and Fig. S9A), as well as at the mRNA level (Fig. S7C). Knockdown of IGF2BP2 elevated the mRNA expression of *IL-1β* and *IL-6*, along with the increased protein expression of iNOS and TNF-α in RA-FLSs (Fig. 4C, D, Fig. S7D and Fig. S9B), as well as an enhanced ability of cell migration and invasion (Fig. 4E, F). Then, *IGF2BP2* overexpressive lentivirus was used to infect RA-FLSs, rescuing the reduced IGF2BP2 expression induced by LPS, both at the protein level (Fig. 4G and Fig. S8C) and mRNA level (Fig. S10A). IGF2BP2 overexpression abrogated the increase in mRNA expression of *IL-1β* and *IL-6* (Fig. 4H, I), as well as the protein expression of iNOS and TNF-α in LPS-exposed RA-FLSs (Fig. S10B and Fig. S11), concurrently reducing the ability of cell migration and invasion (Fig. 4J, K). These results implied that IGF2BP2 inhibited LPS-induced migration, invasion and inflammation reaction in RA-FLSs.

### IGF2BP2 promoted GSTM5 expression via enhancing its mRNA stability

**Fig. 5:**
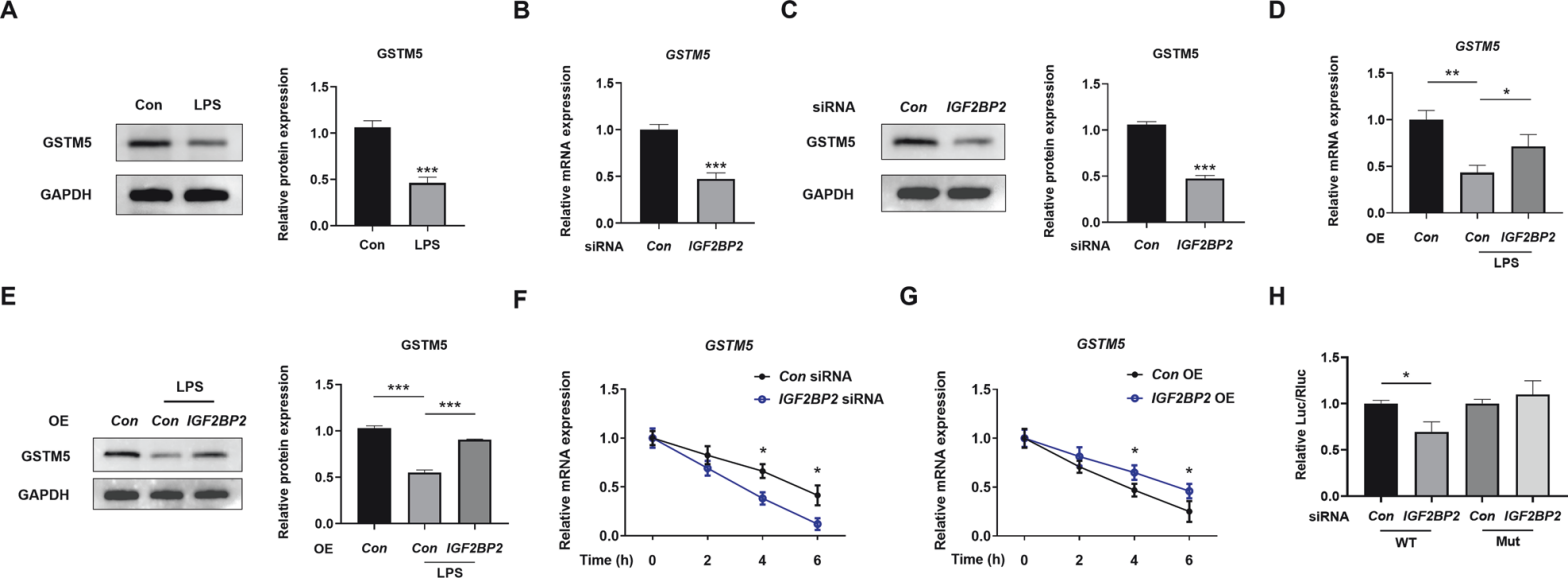
IGF2BP2 promoted GSTM5 expression via enhancing its mRNA stability. **A** Western blotting was used to detect GSTM5 protein level in RA-FLSs after treatment with LPS. **B** qPCR analysis of the mRNA expression of *GSTM5* in RA-FLSs after treatment with *IGF2BP2* siRNA. **C** Western blotting was used to detect GSTM5 protein level in RA-FLSs after treatment with *IGF2BP2* siRNA. **D** qPCR analysis of the mRNA expression of *GSTM5* in LPS-incubated RA-FLSs after treatment with *IGF2BP2* overexpression lentivirus. **E** GSTM5 protein level in LPS-incubated RA-FLSs after overexpressing *IGF2BP2*. **F**, **G** After silencing *IGF2BP2* or overexpressing *IGF2BP2* in RA-FLSs, the mRNA expression of *GSTM5* was analyzed at the predetermined times following actinomycin D (5 μg/mL) treatment. **H** Luciferase assays were performed in 293T cells transfected with WT or mutant luciferase GSTM5 CDS reporters. ^*^P < 0.05, ^**^P < 0.01, ^***^P < 0.001.

Next, we sought to explore the mechanism by which IGF2BP2 regulated GSTM5 in vitro. GSTM5 expression was found to be downregulated in LPS-treated RA-FLSs (Fig. 5A and Fig. S12A). Knockdown of IGF2BP2 inhibited the GSTM5 expression, both at the mRNA level (Fig. 5B) and protein level (Fig. 5C and Fig. S12B). The mRNA expression of *GSTM5* in the LPS group was rescued by IGF2BP2 overexpression in RA-FLSs (Fig. 5D), as well as the protein expression (Fig. 5E and Fig. S12C). Subsequently, RA-FLSs was treated with actinomycin D to inhibit DNA transcription. Knockdown of IGF2BP2 reduced the *GSTM5* mRNA stability at 4-h and 6-h time points (Fig. 5F). Conversely, IGF2BP2 overexpression enhanced *GSTM5* mRNA stability at 4-h and 6-h time points (Fig. 5G). To explore whether this effect depends on m^6^A modification, the expression of METTL3 and METTL14, the key elements of RNA methyltransferase, was decreased in FLSs with the treatment of *METTL3/14* siRNAs (Fig. S13A and Fig. S14). Moreover, the knockdown of METTL3/14 reduced the m^6^A level of total RNAs (Fig. S13B) and inhibited the effect of IGF2BP2 overexpression on *GSTM5* mRNA stability (Fig. S13C). Sequence analysis via SRAMP, a sequence-based m^6^A modification site predictor, revealed a highly confident m^6^A site in *GSTM5* mRNA (Fig. S15 and Table S3). Luciferase reporter assays demonstrated that the knockdown of IGF2BP2 repressed the ratio of firefly luciferase/renilla luciferase in the WT group, with no significant difference observed in the mutated group (Fig. 5H). These results hinted that IGF2BP2 promoted *GSTM5* mRNA stability, leading to an increase in GSTM5 protein expression.

### IGF2BP2 inhibited RA-FLSs migration, invasion and inflammation via GSTM5

**Fig. 6:**
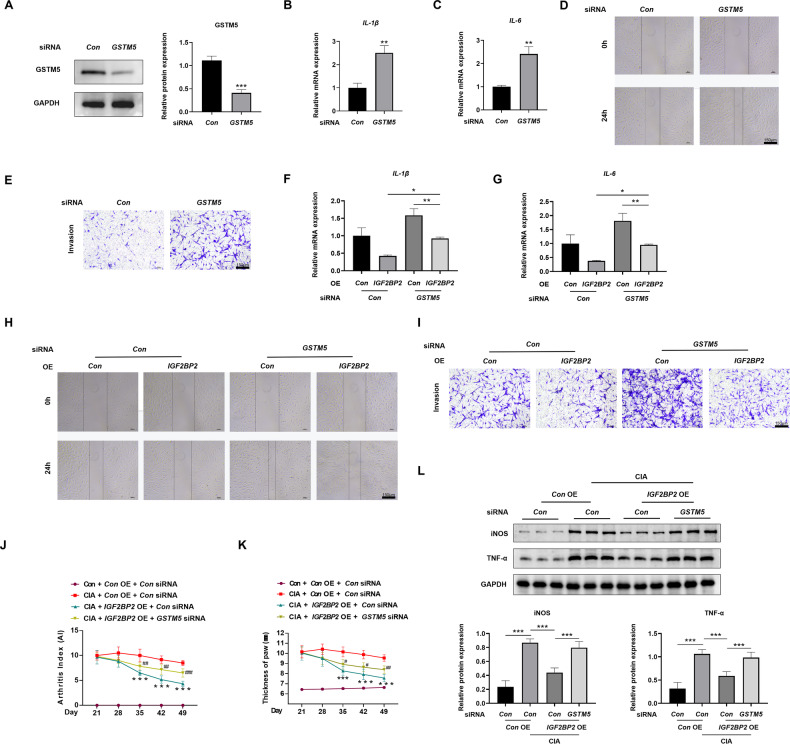
IGF2BP2 inhibited RA-FLSs migration, invasion and inflammation via GSTM5. **A** Western blotting was used to detect GSTM5 protein level in RA-FLSs after treatment with *GSTM5* siRNA. **B**, **C** qPCR analysis of the mRNA expression of *IL-1β* and *IL-6* in RA-FLSs after treatment with *GSTM5* siRNA. **D**, **E** Wound healing and transwell invasion assays showed the capacity of migration and invasion of RA-FLSs after treatment with *GSTM5* siRNA. Scale Bar = 150 µm. **F**, **G** qPCR analysis of the mRNA expression of *IL-1β* and *IL-6* in LPS-incubated RA-FLSs after treatment with *IGF2BP2* overexpression lentivirus or (and) *GSTM5* siRNA. **H**, **I** Wound healing and transwell invasion assays showed the capacity of migration and invasion of LPS-incubated RA-FLSs after treatment with *IGF2BP2* overexpression lentivirus or (and) *GSTM5* siRNA. Scale Bar = 150 µm. The arthritis index (**J**) and paw thickness (**K**) were scored and recorded every 7 days in a blinded manner in different treatment groups. ^*^Compared with CIA; ^#^Compared with CIA + *IGF2BP2* AAV. **L** Western blotting was used to detect TNF-α and iNOS protein levels in synovial tissues of rats in different treatment groups (*n* = 6 per group). ^*^P < 0.05, ^**^P < 0.01, ^***^P < 0.001; ^#^*P* < 0.05^, ##^*P* < 0.01, ^###^*P* < 0.001.

SiRNAs targeted *GSTM5* mRNA were designed to downregulate GSTM5, and *GSTM5* siRNA3 demonstrated the most knockdown efficiency on GSTM5 protein (Fig. S16A and Fig. S17A). Consequently, *GSTM5* siRNA3 was employed to assess GSTM5’s biological function. *GSTM5* siRNA significantly reduced GSTM5 protein expression **(**Fig. 6A and Fig. S18A), as well as *GSTM5* mRNA levels (Fig. S16B). Knockdown of GSTM5 elevated the mRNA expression of *IL-1β* and *IL-6* in RA-FLSs (Fig. 6B, C), while also elevated the protein expression of iNOS and TNF-α (Fig. S16C and Fig. S17B). Additionally, it enhanced the migration and invasion of RA-FLSs (Fig. 6D, E). Knockdown of GSTM5 rescued the decrease in *IL-1β* and *IL-6* mRNA expression (Fig. 6F, G), as well as the reduction in iNOS and TNF-α expression caused by the overexpression of IGF2BP2 in RA-FLSs (Fig. S19 and Fig. S20). Furthermore, knockdown of GSTM5 counteracted the diminished migration and invasion induced by IGF2BP2 overexpression in RA-FLSs (Fig. 6H, I). In vivo experiments indicate that knockdown of GSTM5 after IGF2BP2 overexpression leads to a slow recovery of both arthritis scores and paw thickness (Fig. 6J, K and Fig. S21A, B), and also inhibits the decrease in the expression of iNOS and TNF-α induced by IGF2BP2 overexpression in synovial tissues of CIA rats (Fig. 6L and Fig. S18B). These results indicated that IGF2BP2 restrained RA-FLSs migration, invasion and inflammation via GSTM5.

### IGF2BP2 regulated the expression of MMP9 and MMP13 via GSTM5

**Fig. 7:**
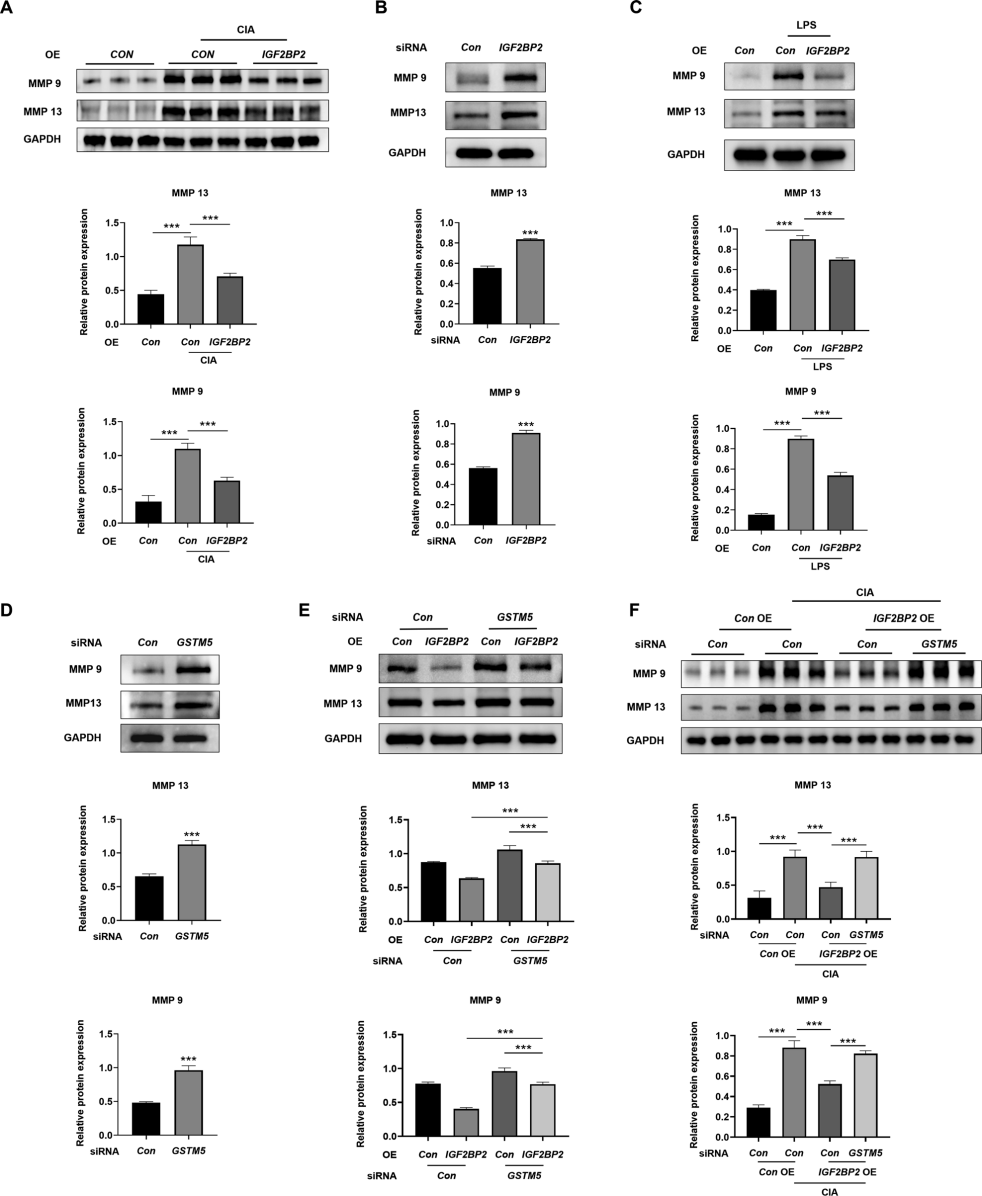
IGF2BP2 regulated the expression of MMP9 and MMP13 via GSTM5. **A** Western blotting was used to detect MMP9 and MMP13 protein levels in synovial tissues of rats after *IGF2BP2* AAV treatment (*n* = 6 per group). **B** Western blotting was used to detect MMP9 and MMP13 protein levels in RA-FLSs after treatment with *IGF2BP2* siRNA. **C** Western blotting was used to detect MMP9 and MMP13 protein levels in LPS-incubated RA-FLSs after overexpressing *IGF2BP2*. **D** Western blotting was used to detect MMP9 and MMP13 protein levels in RA-FLSs after treatment with *GSTM5* siRNA. **E** Western blotting was used to detect MMP9 and MMP13 protein levels in LPS-incubated RA-FLSs after treatment with *IGF2BP2* overexpression lentivirus or (and) *GSTM5* siRNA. **F** Western blotting was used to detect MMP9 and MMP13 protein levels in synovial tissues of rats in different treatment groups (*n* = 6 per group). ^***^P < 0.001.

Given the potential involvement of IGF2BP2 in CIA rats and activated RA-FLSs, we next explored its role in MMPs. The expression of MMP9 and MMP13 was elevated in the synovial tissues of CIA rats, and this elevation was inhibited by injection with *IGF2BP2* overexpressive AAV (Fig. 7A and Fig. S22A). Knockdown of IGF2BP2 promoted the expression of MMP9 and MMP13 in RA-FLSs (Fig. 7B and Fig. S22B). Conversely, IGF2BP2 overexpression suppressed the expression of MMP9 and MMP13 in RA-FLSs (Fig. 7C and Fig. S22C). Similarly, the knockdown of GSTM5 promoted the expression of MMP9 and MMP13 in RA-FLSs (Fig. 7D and Fig. S22D). To verify the role of IGF2BP2-GSTM5 axis in the expression of MMP9 and MMP13, a rescue experiment was performed in RA-FLSs with the treatment of *IGF2BP2* overexpressive lentivirus and *GSTM5* siRNA. Knockdown of GSTM5 significantly inhibited the decrease in the expression of MMP9 and MMP13 induced by IGF2BP2 overexpression, both in RA-FLSs and in synovial tissues of CIA rats (Fig. 7E, F and Fig. S22E, F). These results indicated that IGF2BP2 repressed the expression of MMP9 and MMP13 via GSTM5 both in vitro and in vivo.

## Discussion

The treatment of RA is a complex process aimed at protecting the function of joints and muscles as much as possible, ultimately achieving the goal of complete remission or reducing disease activity. Inhibiting joint inflammatory response to retard lesion development and irreversible bone destruction may be a good strategy for RA treatment [17]. RA is a chronic immune-mediated arthritis that causes the immune system to attack its own joints, and synovitis is a key pathological basis for RA. Abnormal migration and invasion of FLSs are an important manifestation of the progress of synovitis. The synovium of patients with RA transforms into hyperplastic invasive tissue, which destroys the morphology and structure of cartilages and bones. Pathogenic mediators produced by FLSs in the synovial lining contribute to the progression of RA, causing aggressive phenotypes [18]. Therefore, it is particularly important to explore the molecular mechanism of the inflammatory response of FLSs in RA. In this study, IGF2BP2 was decreased in the synovial tissues of RA patients and CIA rats. We demonstrated that IGF2BP2 overexpression alleviated FLS and synovial inflammation, and inhibited cartilage erosion of CIA rats, which provided a new mechanism of FLS dysfunction in RA.

The CIA rat model utilized in our study is widely acknowledged for its resemblance to human RA in terms of pathophysiology, encompassing synovitis, pannus formation, and the degradation of cartilage and bone. This model proves particularly valuable for investigating the immunological facets of RA, as it emulates the autoimmune response observed in human RA [19]. Nevertheless, it is important to acknowledge certain limitations. The CIA model primarily concentrates on the autoimmune response and may not entirely replicate other elements of human RA [20]. For instance, the joint pathology observed in RA is characterized by chronicity and symmetry, while the induced CIA in animals exhibits asymmetry and transience. Moreover, it has been established that the manifestations of arthritis in animal models of CIA exhibit a spontaneous resolution within a defined temporal window, lasting approximately 60 days in mice and approximately 40 days in rats. This temporal aspect holds considerable importance when determining the optimal timing for the introduction of novel therapeutic agents [21–23]. The adjuvant-induced arthritis (AIA) model offers valuable insights, particularly regarding environmental triggers and the innate immune response, while the human TNF transgenic mouse model holds particular relevance for investigating the genetic aspects and systemic inflammation of RA [24]. In summary, the utilization of the CIA rat model yields significant insights into the autoimmune components of RA; However, the inclusion of supplementary models such as AIA and TNF transgenic mice may contribute to a more comprehensive comprehension of the disease and enhance the robustness of our research findings.

Previous studies have shed light on a strong link between IGF2BP2 and chronic inflammation in human disease [13–15]. The proinflammatory or anti-inflammatory role of IGF2BP2 in diseases is still controversial. IGF2BP2 deficient mice are resistant to autoantibody-induced glomerulonephritis and IGF2BP2 deletion initiated only after glomerulonephritis ameliorated disease [13]. In central nervous system, reduced CCL2 production and inflammatory reaction were observed in IGF2BP2 deficient mice. Moreover, IGF2BP2 deletion after experimental autoimmune encephalomyelitis onset was sufficient to mitigate disease severity [14]. These studies suggest that IGF2BP2 deficiency represse the progression in inflammatory-related diseases, which contradicts our research findings. This seemingly contradictory results may be due to the timing of IGF2BP2 intervention. Interestingly, significant histological inflammatory cell infiltrates were observed in the mice with hematopoietic IGF2BP2 deficiency compared to the mice with hematopoietic IGF2BP2 expression. While, cockroach allergen-induced lung inflammation is alleviated by hematopoietic cells lacking IGF2BP2 [15]. This study demonstrated the diversity of inflammatory effects of IGF2BP2 in different organs and tissues. Thus, IGF2BP2 has significant differences in the regulation of inflammation related diseases and cell function, which may be related to different models and cell lines. A clinical study suggested that IGF2BP2 expression was decreased in Crohn’s disease tissues and ulcerative colitis tissues [25]. Moreover, IGF2BP2 expression was reduced in all models of acute inflammation despite not reaching statistical significance [26]. In our study, IGF2BP2 expression was decreased in the synovial tissues of RA patients and CIA rats. IGF2BP2 overexpression alleviated synovial hyperplasia and cartilage destruction in CIA rats and inhibited RA-FLSs migration and invasion. Recent study showed that IGF2BP2 overexpression inhibited the migration and invasion of clear cell renal cell carcinoma [27], which supports our results in this study. Also, IGF2BP2 knockdown increased intracellular reactive oxygen species content in cervical cancer cells [28], which indirectly supports our research finding that IGF2BP2 knockdown induced RA-FLSs inflammation. Given the diversified biological role of IGF2BP2, exploring its downstream mechanisms is an important issue in this study.

There have been reports that RNA m^6^A methylation, as one of the important contents of epigenetics research, performs important functions that affect disease and normal life activities [29, 30]. It must be identified by variable readers in order to exert different downstream effects on the m^6^A group. As a type of m^6^A modification readers, IGF2BP2 enhances the stability of target mRNAs that are modified by RNA methyltransferase in adenosines [31]. In our study, we identified *GSTM5* mRNA as a potential target mRNA of IGF2BP2. IGF2BP2 overexpression significantly promoted the stability of *GSTM5* mRNA and IGF2BP2 knockdown accelerated *GSTM5* mRNA degradation. METTL3 and METTL14 are the key elements of RNA methyltransferase, which promotes the m^6^A modification of RNA in mammals. Knockdown of METTL3/14 reduced the m^6^A level of total RNAs and inhibited the effect of IGF2BP2 overexpression on *GSTM5* mRNA stability. We further directly confirmed the m^6^A position that IGF2BP2 affects the stability of *GSTM5* mRNA by luciferase report gene assay. These results suggested that *GSTM5* mRNA was regulated by IGF2BP2 depending on m^6^A modificated sites. GSTM5 is a member of glutathione S-transferase gene family that is one of the major xenobiotic detoxifying enzymes protecting cells from toxic drugs and environmental electrophiles [32]. Multiple studies have shown that GSTM5 participates in various disease processes by influencing cellular oxidative stress and inflammatory response [33, 34]. However, the role of GSTM5 in joint-related diseases is still unclear. The expression of GSTM5 in the synovial tissues of RA and CIA rats was decreased. Consistent with in vivo experimental results, GSTM5 expression was decreased in LPS-exposed RA-FLSs. This indicated that RA-FLSs lacked GSTM5 in an inflammatory state. Similarly, GSTM5 knockdown acted as a proinflammatory role in RA-FLSs due to elevated mRNA expressions of *IL-1β* and *IL-6*, as well as stronger capability of migration and invasion. Bioinformatics research have revealed the potential of GSTM5 in cell proliferation, migration, and inflammation-related pathways in gastric cancer [35], which also supports our results to some extent. Moreover, GSTM5 also involves in the regulation of cell proliferation, migration and invasion in bladder cancer, lung adenocarcinoma and colorectal cancer [32, 36, 37]. Based on the above facts, GSTM5 affected FLSs function in a way that regulates inflammation, ultimately leading to RA.

MMPs are one type of enzyme that exists in the dermis and are specifically responsible for the decomposition of collagen, which is an important cause of RA [38]. A large number of free radicals caused by inflammatory reaction will indirectly increase the expression of MMP metalloproteinase in the dermis [39]. In this study, IGF2BP2 overexpression inhibited the elevated expressions of MMP9 and MMP13 in the synovial tissues of CIA rats. Moreover, decreased expressions of MMP9 and MMP13 in RA-FLSs were rescued by *GSTM5* siRNA transfection. These results draw a conclusion that is consistent with previous research. Additionally, existing research has shown that MMP9 overexpression promotes inflammation outbreak accompanied by osteoclast formation [40]. Therefore, there is reason to believe that MMP9 and MMP13 can exacerbate FLSs inflammation caused by IGF2BP2 deficiency in RA.

## Materials and methods

### Tissues and cell culture

Synovial tissues were taken from osteoarthritis (OA) and RA patients underwent artificial total knee joint replacement at the Orthopedic Department of the Affiliated Hospital of Nantong University. All patients participating in this experiment must submit informed consent. The study plan was approved by the Ethics Committee of the Affiliated Hospital of Nantong University (2020-L136). All enrolled patients with RA met the American College of Rheumatology (ACR)/European League Against Rheumatism 2010 classification criteria. RA synovial tissue fragments were digested in DMEM-F12 with type II collagenase (1 mg/mL) for 1-2 h and shaking culture at 37 °C for 2 h. The RA fibroblast-like synoviocytes (RA-FLSs) were collected with centrifugation and cultured in 90% DMEM-F12 medium with 10% FBS and 1% penicillin-streptomycin at 37 °C in 5% CO_2_/95% air. In this study, we used 3-6 passage cells. When RA-FLSs reached 70-80% confluence, 1 μg/mL lipopolysaccharide (LPS) (HY-D1056, MCE, USA) was used to stimulate the cells for 24 h to mimic the pathological features of RA in vitro as a cellular model.

### Cell transfection

The small interfering RNAs (siRNAs) of *IGF2BP2*, *GSTM5*, *METTL3* and *METTL14* were obtained from GenePharma (Shanghai, China). The sequences are as follows: si-*IGF2BP2*-1 sense: 5′-GCGAAAGGAUGGUCAUCAUTT-3′; si-*IGF2BP2*-2 sense: 5′-GCUGUUAACCAACAAGCCATT-3′; si-*IGF2BP2*-3 sense: 5′-ACAGGACUGUCCGUGCUAUTT-3′; si-*GSTM5*-1 sense: 5′-GCUGGUCAGACUGUGCUAUTT-3′; si-*GSTM5*-2 sense: 5′-GGAUUCCUUGCCUAUGAUTT-3′; si-*GSTM5*-3 sense: 5’-GGGUUUGAAGAAGAUCUCUTT-3′; si-*GSTM5*-4 sense: 5′-GAAAGUCAGCUACAUGGAATT-3′; si-*METTL3* sense: 5′-GCUGCACUUCAGACGAAUUTT-3′; si-*METTL14* sense: 5′-GCAGCACCUCGAUCAUUUATT-3′. The siRNAs were transfected into RA-FLSs using a transfection reagent according to the manufacturer’s recommendations. At 6 h after transfection, the medium was replaced with a complete medium. The cells were collected for subsequent experiments at 48 h post-transfection.

### Cell infection

The lentiviral constructs carrying *IGF2BP2* were provided by Miaoling Biology (Wuhan, China). The cells were planted in a 6-well plate at a density of 50–60%, and 80 μL lentiviral constructs, 7.5 μL polybrene (Miaoling Biology, Wuhan, China) and 1 mL basal medium were added sequentially. After overnight cultivation, 1 mL complete medium was added for further culture.

### Western blotting

Cells were lysed with radioimmunoprecipitation assay (RIPA) buffer containing 1% protease and phosphatase inhibitor cocktail (WB3100, New Cell & Molecular Biotech, Suzhou, China). The proteins were loaded on SDS-PAGE gels, and then transferred onto polyvinylidene fluoride (PVDF) membrane (03010040001, Roche Diagnostics Gmbh, Germany). After blocking with NcmBlot blocking buffer (P30500, New Cell & Molecular Biotech, Suzhou, China) for 10 min at room temperature, the membranes were incubated with the primary antibody against the following targets: IGF2BP2 (1:2000, 11601-1-AP, Proteintech, Wuhan, China), GSTM5 (1:4000, 14502-1-AP, Proteintech, Wuhan, China), MMP9 (1:2000, ab76003, Abcam, England), MMP13 (1:1000, 18165-1-AP, Proteintech, Wuhan, China), iNOS (1:1000, 18985-1-AP, Proteintech, Wuhan, China), TNF-α (1:1000, 60291-1-Ig, Proteintech, Wuhan, China), METTL3 (1:1000, 15073-1-AP, Proteintech, Wuhan, China), METTL14 (1:1000, 26158-1-AP, Proteintech, Wuhan, China) and GAPDH (1:10000, 60004-1-Ig, Proteintech, Wuhan, China) overnight at 4 °C. After washing three times with Tris-buffered saline (TBS) containing 1% Tween-20 (TBST), the membranes were incubated with secondary antibodies for 1 h at room temperature. The protein bands were visualized using an enhanced chemiluminescent detection system (P10300, New Cell & Molecular Biotech, Suzhou, China). ImageJ software was performed for quantitative analysis.

### Immunohistochemistry (IHC) staining

Synovial tissue samples were fixed in 4% paraformaldehyde, dehydrated, and subsequently embedded in paraffin. The paraffin blocks were cut into 5 μm slices by a microtome. The sections were prepared by these procedures of xylene dewaxing, gradient ethanol hydration and endogenous peroxidase blocking. Then, the sections were incubated at room temperature with the QuickBlock™ immunostaining blocking solution (P0260, Beyotime Biotech, Shanghai, China) for 10 min. Primary antibodies for IGF2BP2 (1:100, 11601-1-AP, Proteintech, Wuhan, China) and GSTM5 (1:100, 14502-1-AP, Proteintech, Wuhan, China) were applied to the sections overnight at 4 °C. After flushing with PBS, the sections were incubated with horseradish peroxidase-conjugated secondary antibody at 37 °C for 20 min and diaminobenzidine (DAB) chromogen at room temperature for 5-8 min (PV-9000, ZLI-9018, ZSGB-BIO, Beijing, China). Immediately, the sections were counterstained with hematoxylin staining solution for 20 s and differentiated in 1% hydrochloric acid ethanol for 20 s. The sections were finally dehydrated in an ascending series of ethanol, cleared in xylene, sealed with neutral gum and examined using Leica light microscope.

### Immunofluorescent staining

The sections were permeabilized with 0.1% Triton X-100 in PBS for 15 min and then blocked with 5% bovine serum albumin (BSA) for 1 h at room temperature. the sections were incubated overnight with primary antibodies IGF2BP2 (1:200, 11601-1-AP, Proteintech, Wuhan, China), GSTM5 (1:100, 14502-1-AP, Proteintech, Wuhan, China) and Vimentin (1:100; AF0318, Beyotime Biotech, Shanghai, China). On the next day, a mixture of Alexa Fluor 488 (1:500; A0423, Beyotime Biotech, Shanghai, China) and Alexa Fluor 555-labeled (1:500; A0460, Beyotime Biotech, Shanghai, China) secondary antibodies were added in a dark room and incubated for 2 h at room temperature. The stained sections were examined with Olympus fluorescence microscope.

### Rat models of arthritis

Six-week-old Wistar male rats were obtained from the Experimental Animal Center of Nantong University. All the animal experimental protocols were approved by the Animal Ethics Committee of Nantong University (Permit Number: S20200323-124). The rats were fed in a specific pathogen-free (SPF) facility for two weeks. Wistar rats were randomly divided into four groups (n = 6 per group): Con, CIA, CIA + *IGF2BP2* AAV, and CIA + *IGF2BP2* AAV + *GSTM5* siRNA. No blinding was done in the animal experiment. A CIA model was established in Wistar rats as an RA animal model. Commonly, the incomplete Freund’s adjuvant (7002, Chondrex, USA) and bovine type II collagen (20022, Chondrex, USA) were mixed at a ratio of 1:1. Then, CIA was elicited by injection intradermally with 200 µL mixture at the base of the tail on day 1, and the 100 µL of immune enhancer was boosted on day 7. Twenty-one days later, the severity of arthritis was evaluated by two independent and blinded observers using an arthritis index: 0 points for no swelling, 1 point for 1 or 2 interphalangeal joint involvement, 2 points for joint and toe mild swelling scored, 3 points for toe and ankle joint swelling, and 4 points for full paw severe arthritis. This resulted in a maximum possible score of 16 per limb (maximum score of 4 each for the limb). The CIA model was made successfully with a total points score beyond 6. One day after booster application, the knee and ankle joints were intra-articularly injected with 5×10^10^ vector genomes (vg) of AAV-*IGF2BP2* (Genechem, Shanghai, China). For the CIA + *IGF2BP2* AAV + *GSTM5* siRNA group, 21 days after the booster application, 5 nmol of *GSTM5* siRNA (Ribobio Biotech, Guangzhou, China) were intra-articularly injected into the knee and ankle joints once a week for a total intervention period of three weeks. Each knee or ankle joint was considered independently. After 49 days, the rats were sacrificed. The knee synovial tissues and ankles of the rats were taken for the following experiment.

### Wound healing assay

RA-FLSs were seeded in a 6-well plate, and then the cells were cultured in basal DMEM-F12 medium without serum. A sterile 200 µL pipette tip was used to make a straight scratch on the confluent cell monolayer. Wound closure was captured at 0 and 24 h using an Olympus inverted microscope.

### Cell invasion assay

For the transwell invasion assay, the upper surfaces of transwell inserts were pre-coated with Matrigel. Cells were seeded on the upper chambers with 200 µL of serum-free DMEM-F12 medium at a density of 2 × 10^4^. The lower chambers were filled with 500 µL complete medium. After 24 h of incubation, the cells from the upper chamber were removed, and the invaded cells were fixed with 4% paraformaldehyde and stained with crystal violet. All images were captured by microscopy.

### RNA isolation and quantitative real-time polymerase chain reaction (qPCR)

The total mRNA was extracted by RNA-Quick Purification Kit (ES-RN001, Yishan Biotech, Shanghai, China). The extracted total RNA was reverse transcribed into cDNA using the HiScript III RT SuperMix for qPCR (R323, Vazyme Biotech, Nanjing, China). The qPCR analysis was detected by QuantStudio5 with SYBR (G3326, Servicebio Biotech, Wuhan, China). The expression levels of relative mRNA were obtained by the 2^-ΔΔCT^ method. The sequences of the primers are provided in Table S1.

### Hematoxylin-eosin staining and Safranin-O assay

The ankle samples were fixed with 4% paraformaldehyde for 48 h and made into tissue sections after putting off calcium for 45 days. Each glass slide was stained with H&E or Safranin-O for general histological evaluation.

### Actinomycin D treatment

IGF2BP2 was overexpressed in RA-FLSs by lentivirus infection or knockdown IGF2BP2 by siRNA transfection. Then, the transcription was blocked by adding actinomycin D of 5 μg/mL (GC16866, GlpBio, USA) to the cell culture medium for 0 h, 2 h, 4 h, 6 h and 8 h. Total RNAs were extracted and *IGF2BP2* mRNA expression was detected by qPCR.

### Dual-luciferase reporter assay

293 T cells were seeded in 96-well plates and transfected with pmirGLO luciferase vector with wild-type (GGACU) or mutated sequence (GGTCU) in the coding sequence of *GSTM5* mRNA (RiboBio, China) for 24 h. Reporter assays were performed following the manufacturer’s protocol (E2920, Promega, USA). Renilla luciferase activity was normalized to a firefly luciferase signal and quantified as a percentage of the control.

### Meusurement of total M^6^A

Total m^6^A modification level was measured in 200 ng RNA extracted from RA-FLSs transfected with *METTL3/14* siRNA. M^6^A levels of total RNA were measured using an m^6^A RNA methylation quantification kit (P-9005, EpiGentek, USA) according to the manufacturer’s instructions.

### Statistical analysis

The data were analyzed using SPSS 13.0 software. Two-group and multiple-group differences were evaluated using two-tailed Student’s *t* tests and one-way analysis of variance (F) with Bonferroni’s post hoc tests, respectively. The variance was similar between the groups that were being statistically compared. No samples or animals in this study were excluded from the analysis. All the data are presented in the form of mean ± SD from three independently performed experiments. *P* < 0.05 was considered significant.

### Supplementary information

Supplementary material

## Data Availability

All data is available in the main text or the supplementary materials. All original western blot images are available in the Supplemental Material.
