# Supplementary material for: IGF2BP2 regulates the inflammation of fibroblast-like synoviocytes via GSTM5 in rheumatoid arthritis
Source: Cell Death Discov. 2024 May 3;10:215. doi: 10.1038/s41420-024-01988-3 (PMC11068746; doi:10.1038/s41420-024-01988-3)
Supplement: Supplementary file 1 — Supplementary material [file 41420_2024_1988_MOESM1_ESM.doc]

**Supplementary Information of**

**IGF2BP2 regulates the inflammation of fibroblast-like synoviocytes via GSTM5 in rheumatoid arthritis**

Yunyi Nan^1^, Minhao Chen^1^, Weijie Wu^1, 3^, Rongrong Huang^2^, Weiwei Sun^1^, Qian Lu^4^, Zhifeng Gu^4^, Xingxing Mao^3, *^, Hua Xu^1, *^, Youhua Wang^1, *^

^1^ Department of Orthopaedics, Affiliated Hospital of Nantong University, Medical School of Nantong University, Nantong 226001, China

^2^ Department of Pharmacy, Affiliated Hospital of Nantong University, Nantong 226001, China

^3^ Department of Orthopaedics, Affiliated Nantong Hospital of Shanghai University, The Sixth People’s Hospital of Nantong, Nantong 226001, China

^4^ Department of Rheumatology, Affiliated Hospital of Nantong University, Nantong 226001, China

* Corresponding author: Youhua Wang, Email: [wangyouhua99@163.com](mailto:wangyouhua99@163.com,); Hua Xu, Email: [xuhua1981111@126.com;](mailto:xuhua1981111@126.com;) Xingxing Mao, Email: 15152882182@139.com.

Yunyi Nan, Minhao Chen and Weijie Wu contributed equally to this work and should be considered as equal first authors.

**Table S1. Sequence details of primers in qRT-PCR.**

| Species | Gene | Sequence (5′ -3′) |
| --- | --- | --- |
| Rat  Rat  Rat  Rat | *GAPDH-F*  *GAPDH-R*  *IGF2BP2-F*  *IGF2BP2-R* | GACATGCCGCCTGGAGAAAC  AGCCCAGGATGCCCTTTAGT  TGGCTGAGTATGGGACGGTA  CCTCGAACTGATGCCCACTT |
| Rat  Rat | *IL-1β-F*  *IL-1β-R* | AATCTCACAGCAGCATCTCGACAAG  TCCACGGGCAAGACATAGGTAGC |
| Rat  Rat  Human  Human | *IL-6-F*  *IL-6-R*  *GAPDH-F*  *GAPDH-R* | AGTTGCCTTCTTGGGACTGATGTTG  GGTATCCTCTGTGAAGTCTCCTCTCC  CAGGAGGCATTGCTGATGAT  GAAGGCTGGGGCTCATTT |
| Human | *IGF2BP2-F* | AGCCTGTCACCATCCATGC |
| Human | *IGF2BP2-R* | CTTCGGCTAGTTTGGTCTCATC |
| Human  Human | *IL-1β-F*  *IL-1β-R* | GCCAGTGAAATGATGGCTTATT  AGGAGCACTTCATCTGTTTAGG |
| Human  Human | *IL-6-F*  *IL-6-R* | GACAGCCACTCACCTCTTCAGAAC  GCCTCTTTGCTGCTTTCACACATG |
| Human | *GSTM5-F* | CCATCCTGCGCTACATTGC |
| Human | *GSTM5-R* | CCAGCTCCATGTGGTTATCCAT |
| Rat | *LOC100912008-F* | TGTTCGCTCAGGGATGGTCT |
| Rat | *LOC100912008-R* | CCTTCACTGGCCTGTACTCC |
| Rat | *Tmprss4-F* | AGGACTTGACCAACCCCATAG |
| Rat | *Tmprss4-R* | GATCTGTCACCCAAGACAGCA |
| Rat | *Gstm5-F* | CCCAAAATGTCGTGCTCCAAG |
| Rat | *Gstm5-R* | TAGTCAGGAGCTTCCCCACA |
| Rat | *Arx-F* | TGTCCACGCTCCCGTTTTTA |
| Rat | *Arx-R* | AAGTGGGGTATCCGGAGGAA |

**Table S2. Original CT value of *Tmprss4* and *GAPDH* mRNA in rat synovial tissues.**

| Sample | *GAPDH* | *GAPDH* | *Tmprss4* | *Tmprss4* |
| --- | --- | --- | --- | --- |
| Con1 | 23.06 | 22.82 | Undetermined | Undetermined |
| Con2 | 22.89 | 23.62 | 34.04 | Undetermined |
| Con3 | 22.89 | 22.04 | 33.64 | 32.90 |
| CIA1 | 21.48 | 21.41 | 35.89 | Undetermined |
| CIA2 | 22.98 | 22.74 | Undetermined | Undetermined |
| CIA3 | 20.71 | 20.92 | 33.54 | 32.89 |
| AAV1 | 22.80 | 23.21 | Undetermined | 34.14 |
| AAV2 | 21.65 | 22.07 | 34.22 | 34.78 |
| AAV3 | 22.09 | 21.82 | 33.68 | 33.86 |

**Table S3.** **Prediction of m^6^A sites in *GSTM5* mRNA.**

| # | Position | Sequence | Score  (binary) | Score  (knn) | Score  (spectrum) | Score  (combined) | Decision |
| --- | --- | --- | --- | --- | --- | --- | --- |
| 1 | 88 | GG**A**CA | 0.564 | 0.563 | 0.578 | 0.569 | Low confidence |
| 2 | 136 | AG**A**CU | 0.664 | 0.673 | 0.598 | 0.638 | High confidence |
| 3 | 181 | UG**A**CU | 0.54 | 0.617 | 0.597 | 0.566 | Low confidence |
| 4 | 229 | GG**A**CU | 0.732 | 0.541 | 0.637 | 0.684 | Very high confidence |
| 5 | 355 | GG**A**CA | 0.690 | 0.518 | 0.459 | 0.589 | Moderate confidence |
| 6 | 401 | AG**A**CU | 0.646 | 0.609 | 0.550 | 0.605 | Moderate confidence |
| 7 | 428 | AA**A**CU | 0.580 | 0.291 | 0.633 | 0.587 | Moderate confidence |
| 8 | 452 | GA**A**CU | 0.518 | 0.603 | 0.613 | 0.560 | Low confidence |
| 9 | 610 | GG**A**CU | 0.655 | 0.603 | 0.524 | 0.600 | Moderate confidence |
| 10 | 1176 | GG**A**CU | 0.741 | 0.671 | 0.300 | 0.561 | Low confidence |
| 11 | 1406 | GA**A**CU | 0.64 | 0.721 | 0.495 | 0.586 | Moderate confidence |
| 12 | 1496 | AG**A**CU | 0.644 | 0.630 | 0.476 | 0.576 | Low confidence |

**Supplementary Figures**

**
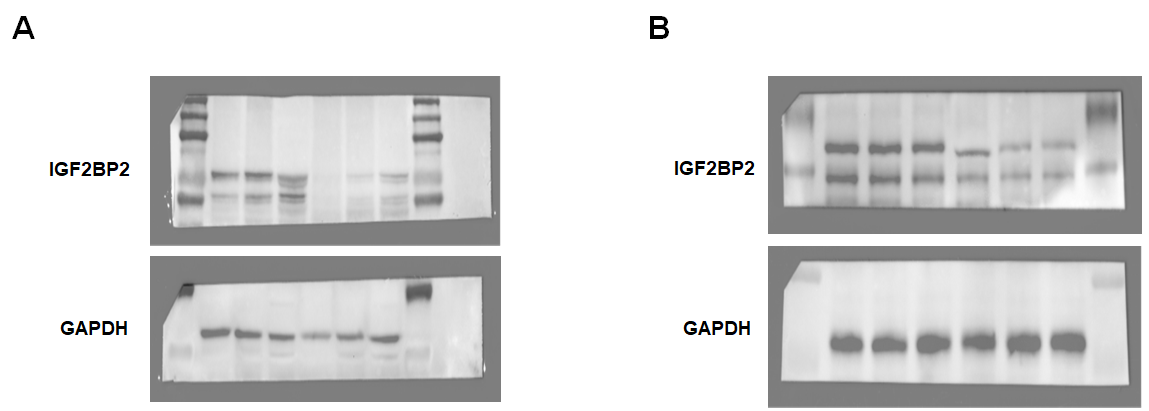
**

**Figure S1. Original blots in Figure 1. (A)** Original blots in Figure 1A. **(B)** Original blots in Figure 1F.


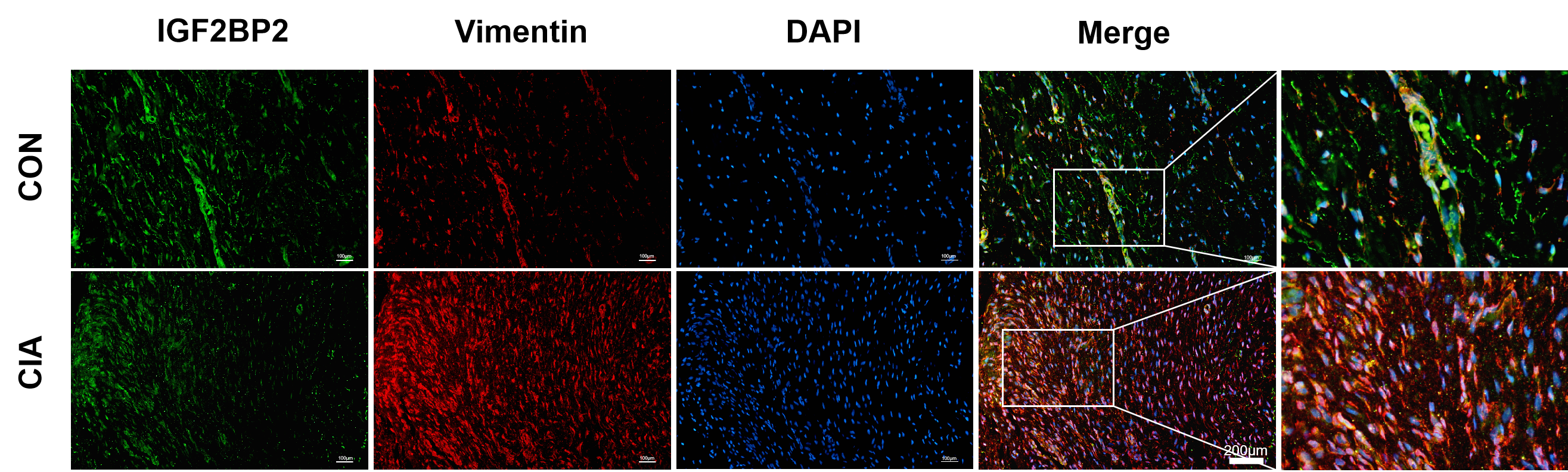


**Figure S2. The co-localization of IGF2BP2 and Vimentin in rat synovial tissues.** Immunofluorescence staining of IGF2BP2 (green) and Vimentin (red) in the control and CIA rat synovial tissues. Scale Bar = 200 µm.


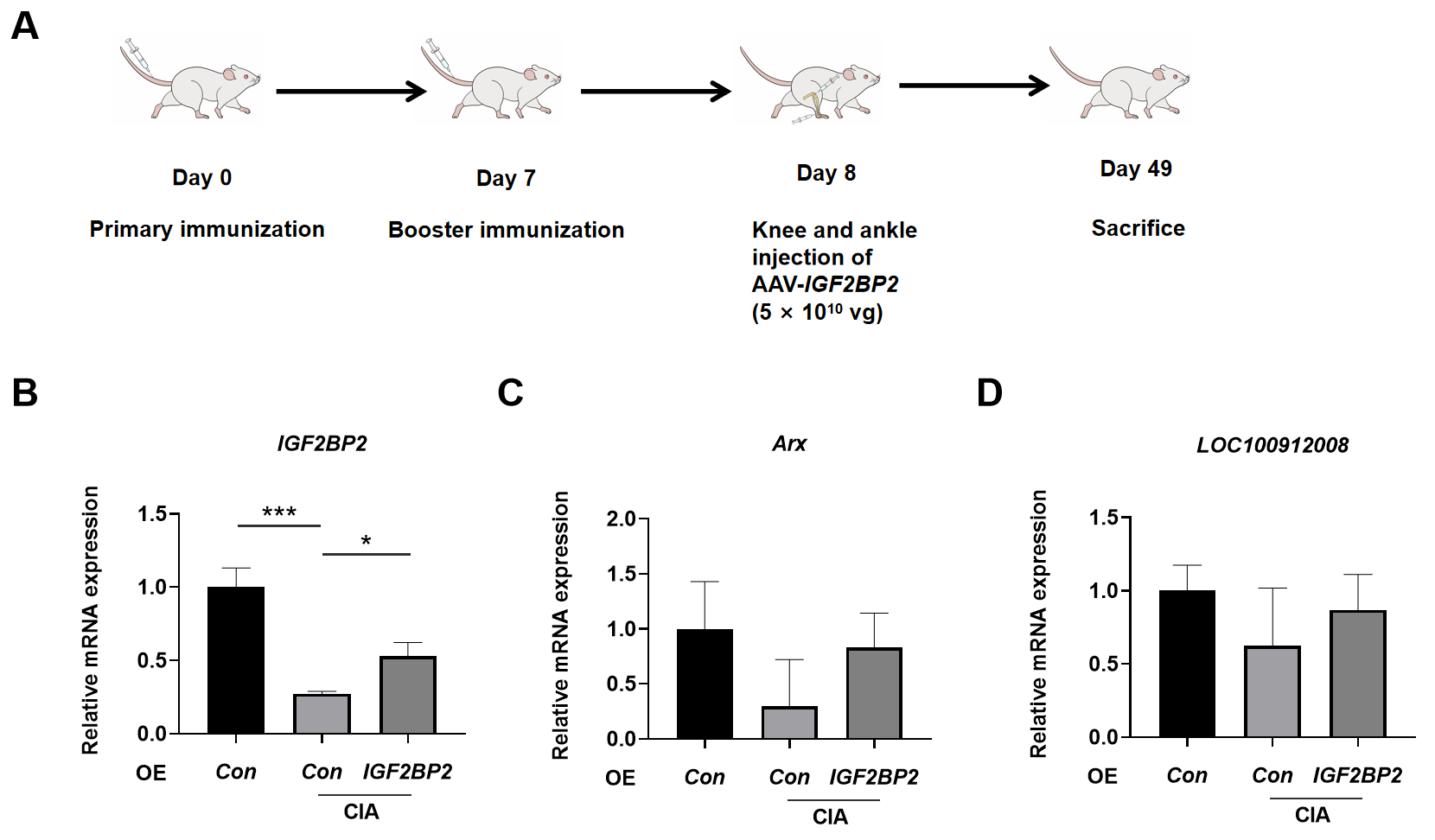


**Figure S3. The effect of IGF2BP2 overexpression on the expression of potential target mRNA in rat synovial tissues. (A)** Timeline of the animal experiment. **(B-D)** The expression of *IGF2BP2* mRNA **(B)**, *Arx* mRNA **(C)** and *LOC100912008* mRNA **(D)** in the control and CIA rat synovial tissues with *IGF2BP2* adeno-associated virus injection. ^*^*P* < 0.05, ^***^*P* < 0.001.


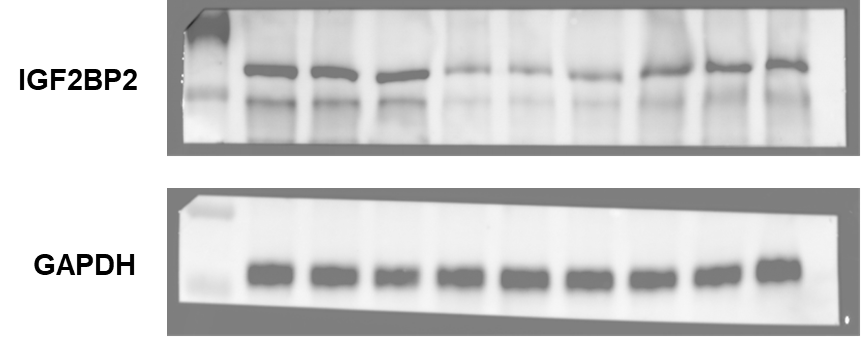


**Figure S4. Original blots in Figure 2.** Original blots in Figure 2A.


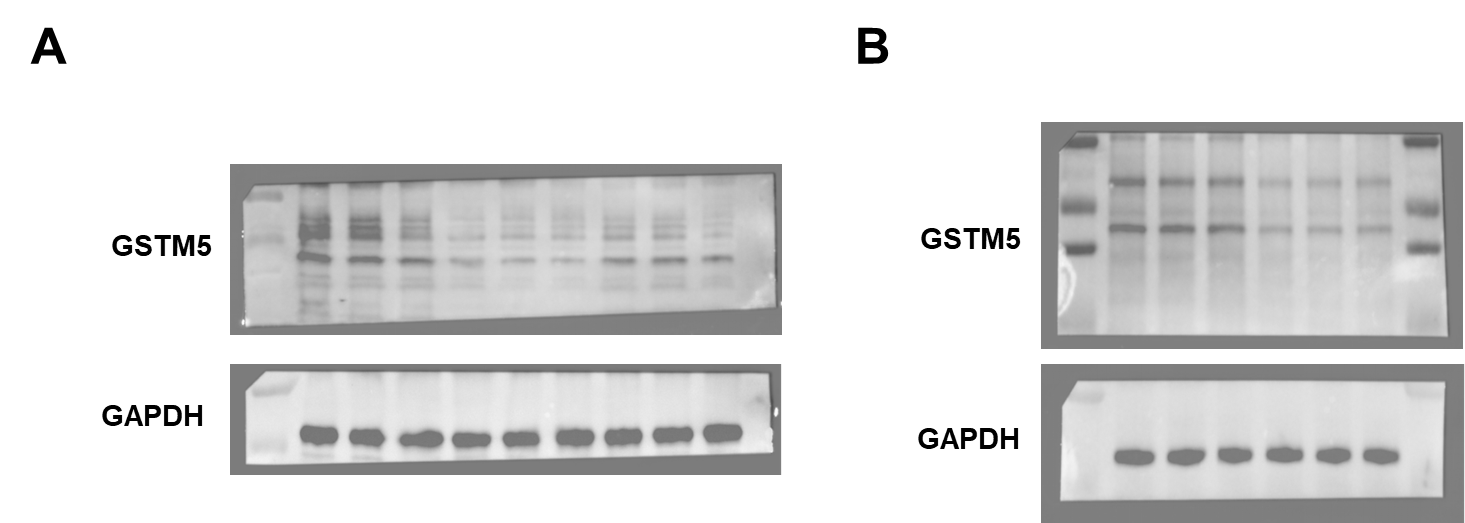


**Figure S5. Original blots in Figure 3. (A)** Original blots in Figure 3E. **(B)** Original blots in Figure 3G.


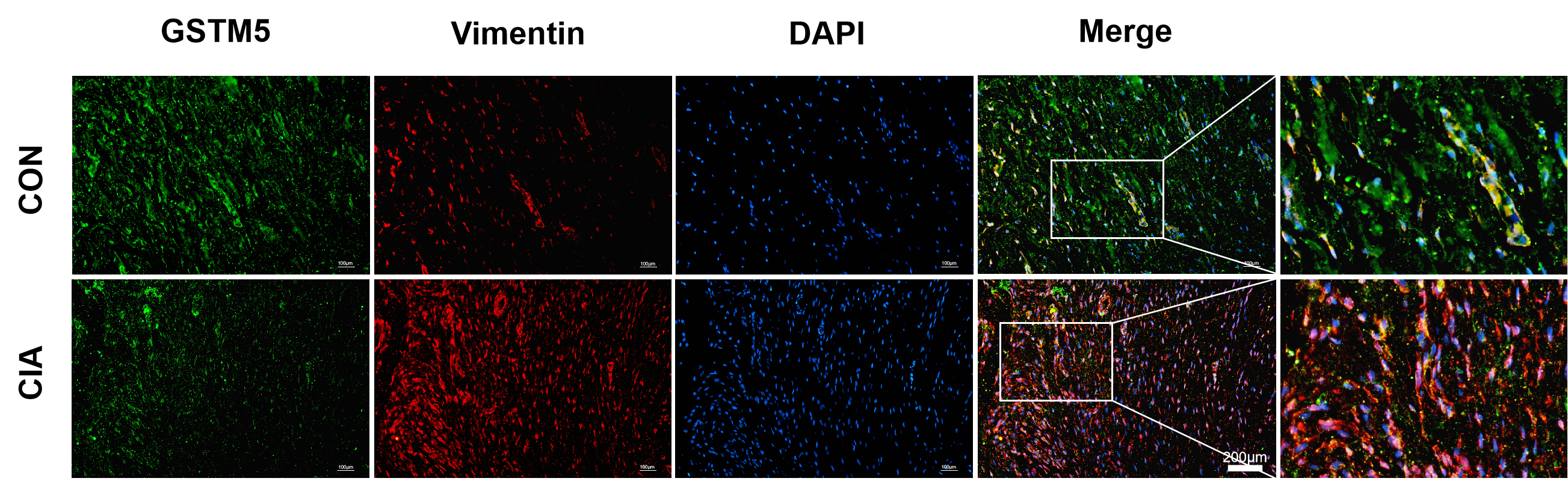


**Figure S6. The co-localization of GSTM5 and Vimentin in rat** **synovial tissues.** Immunofluorescence staining of GSTM5 (green) and Vimentin (red) in the control and CIA rat synovial tissues. Scale Bar = 200 µm.


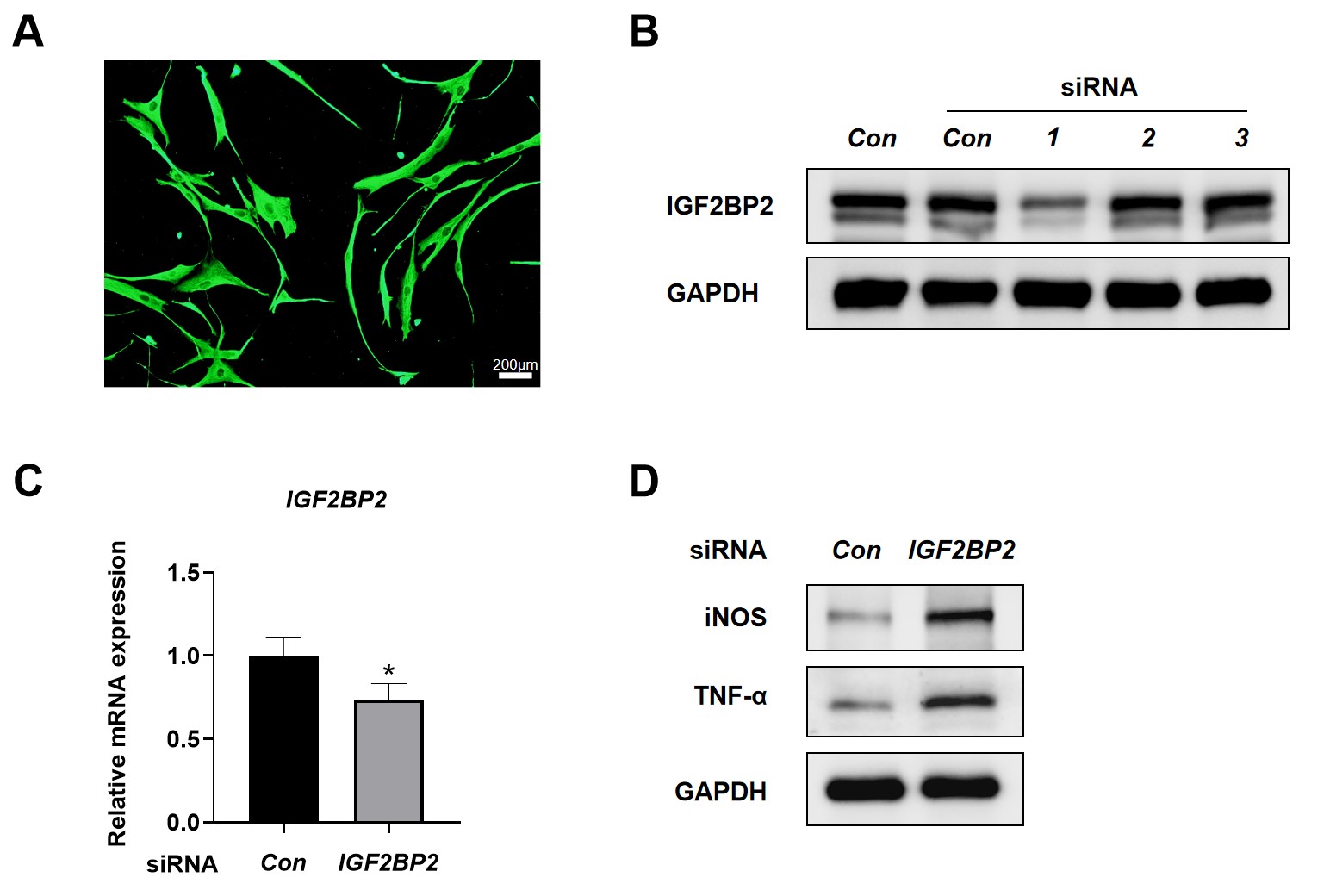


**Figure S7. The effect of *IGF2BP2* siRNA on the inflammatory response in RA-FLSs. (A)** Confirmation of RA-FLSs purity**.** Detection of Vimentin expression by immunofluorescence indicated that the extracted cells were RA-FLSs. Scale Bar = 200 µm. **(B)** Western blotting was used to detect IGF2BP2 protein level in RA-FLSs after treatment with *IGF2BP2* siRNAs. **(C)** The mRNA expression of *IGF2BP2* in RA-FLSs after treatment with *IGF2BP2* siRNA. **(D)** Western blotting was used to detect TNF-α and iNOS protein levels in RA-FLSs after treatment with *IGF2BP2* siRNA. ^*^*P* < 0.05.

**
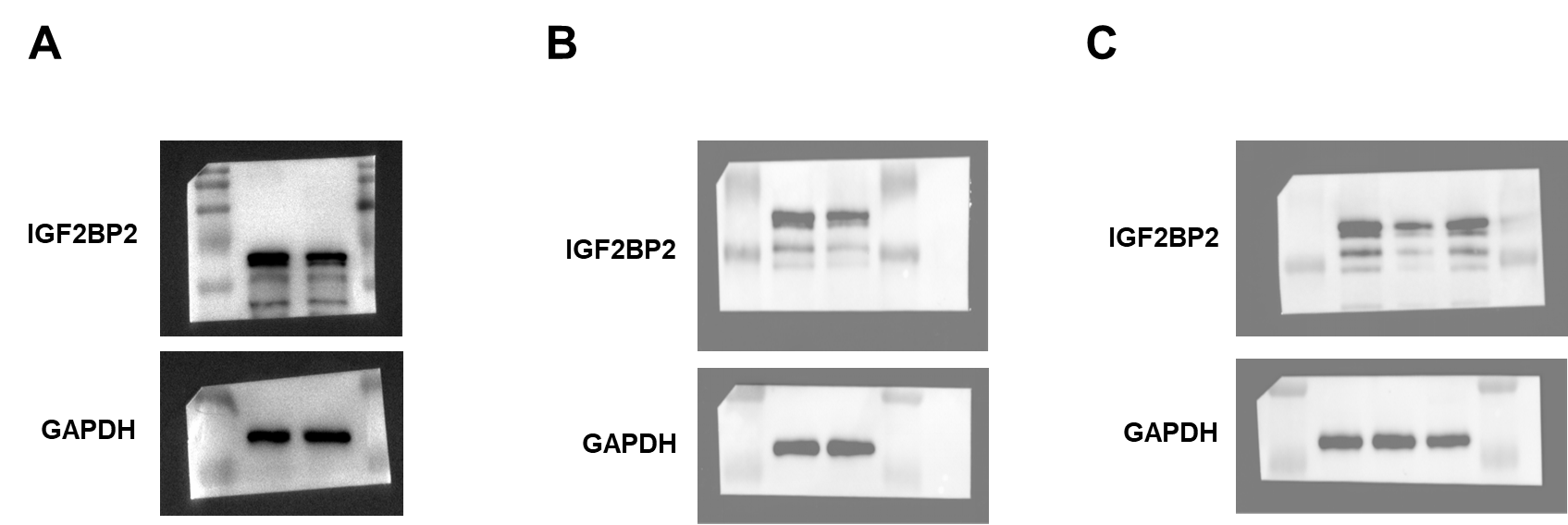
**

**Figure S8. Original blots in Figure 4. (A)** Original blots in Figure 4A. **(B)** Original blots in Figure 4B. **(C)** Original blots in Figure 4G.

**
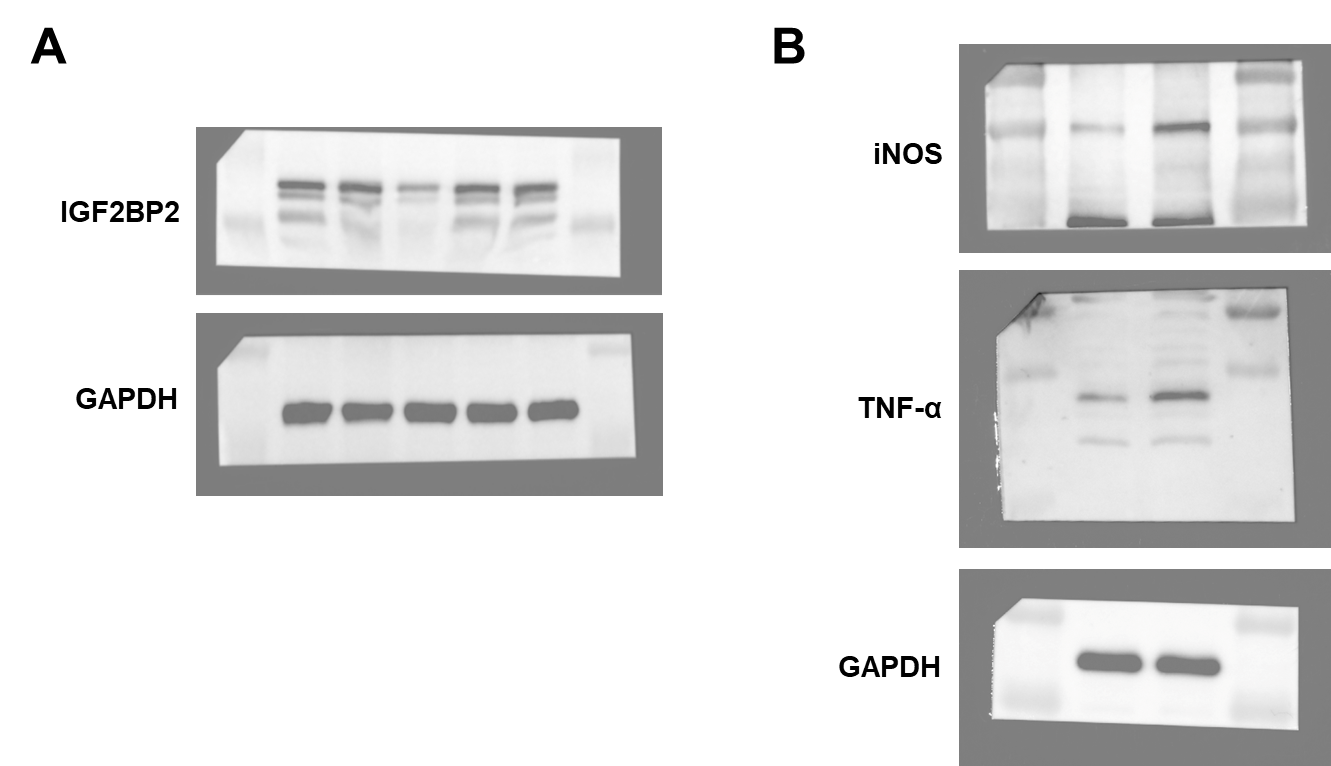
**

**Figure S9. Original blots in Figure S7. (A)** Original blots in Figure S7B. **(B)** Original blots in Figure S7D.


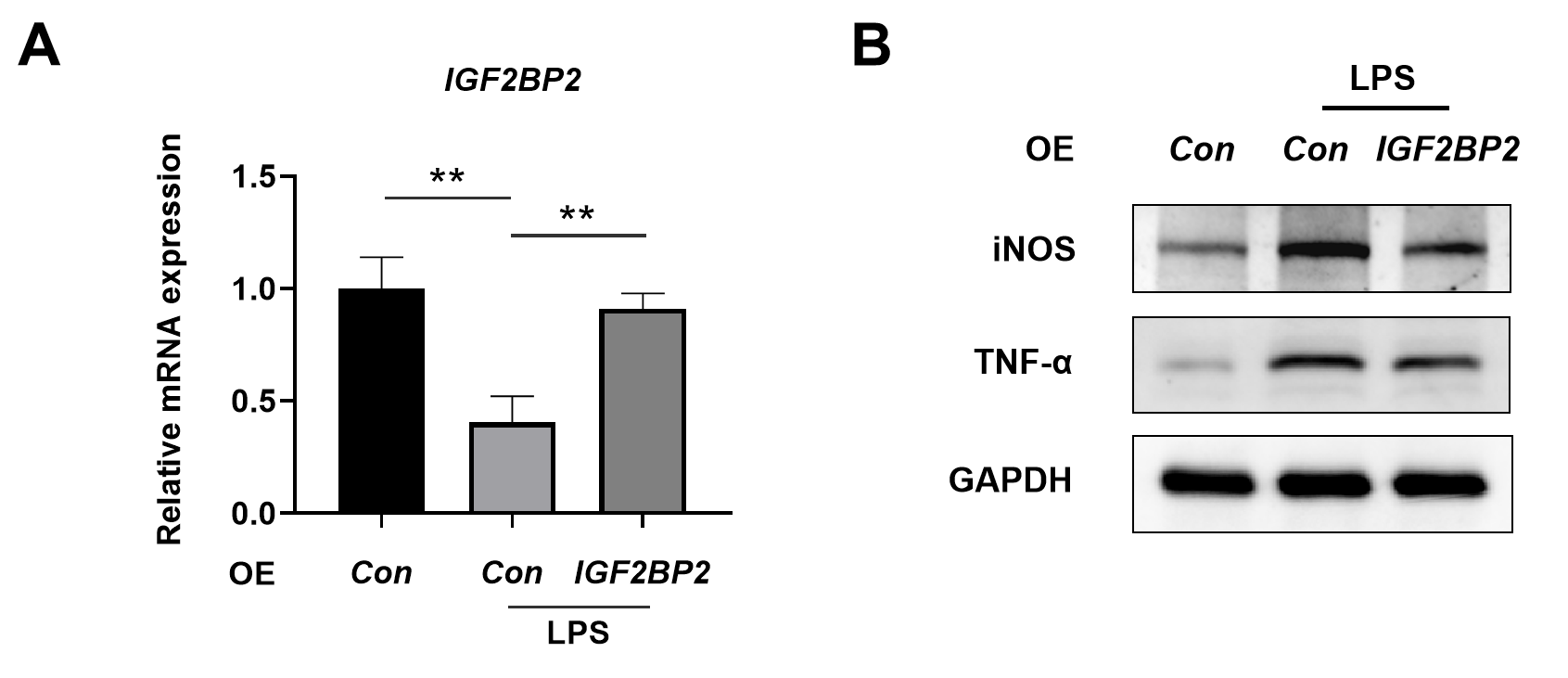


**Figure S10. The effect of IGF2BP2 overexpression on the inflammatory response in RA-FLSs. (A)** The mRNA expression of *IGF2BP2* in LPS-exposed RA-FLSs after treatment with *IGF2BP2* overexpressive lentivirus. **(B)** Western blotting was used to detect TNF-α and iNOS protein levels in RA-FLSs after overexpressing *IGF2BP2*. ^**^*P* < 0.01.


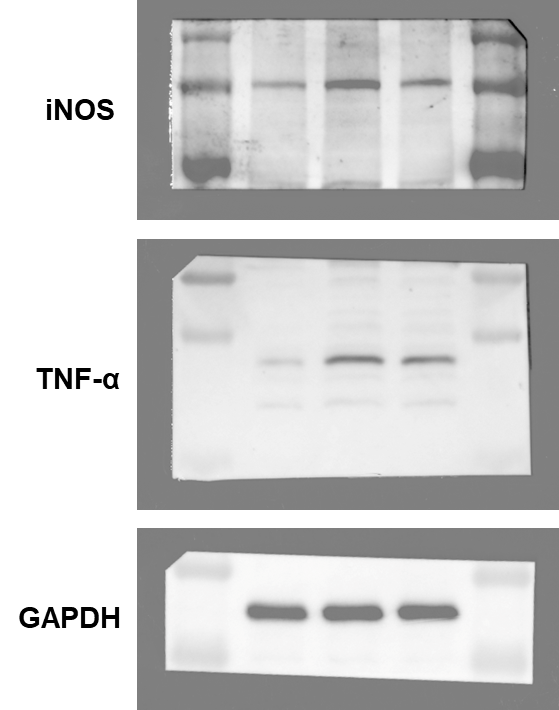


**Figure S11. Original blots in Figure S10.** Original blots in Figure S10B.


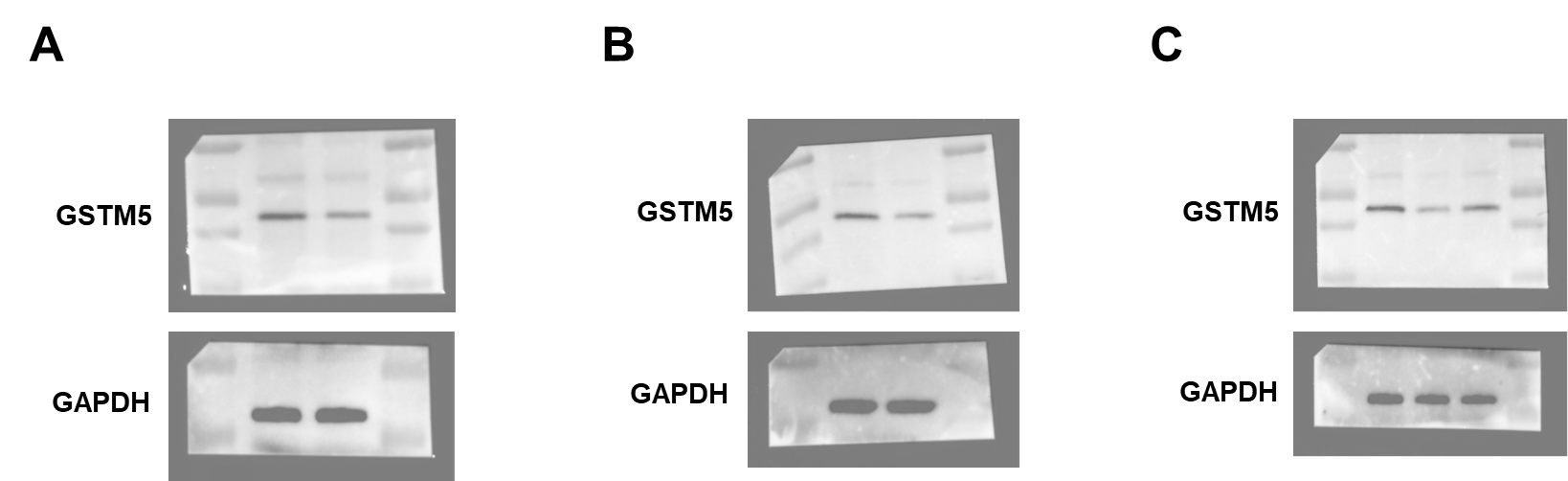


**Figure S12. Original blots in Figure 5. (A)** Original blots in Figure 5A. **(B)** Original blots in Figure 5C. **(C)** Original blots in Figure 5E.


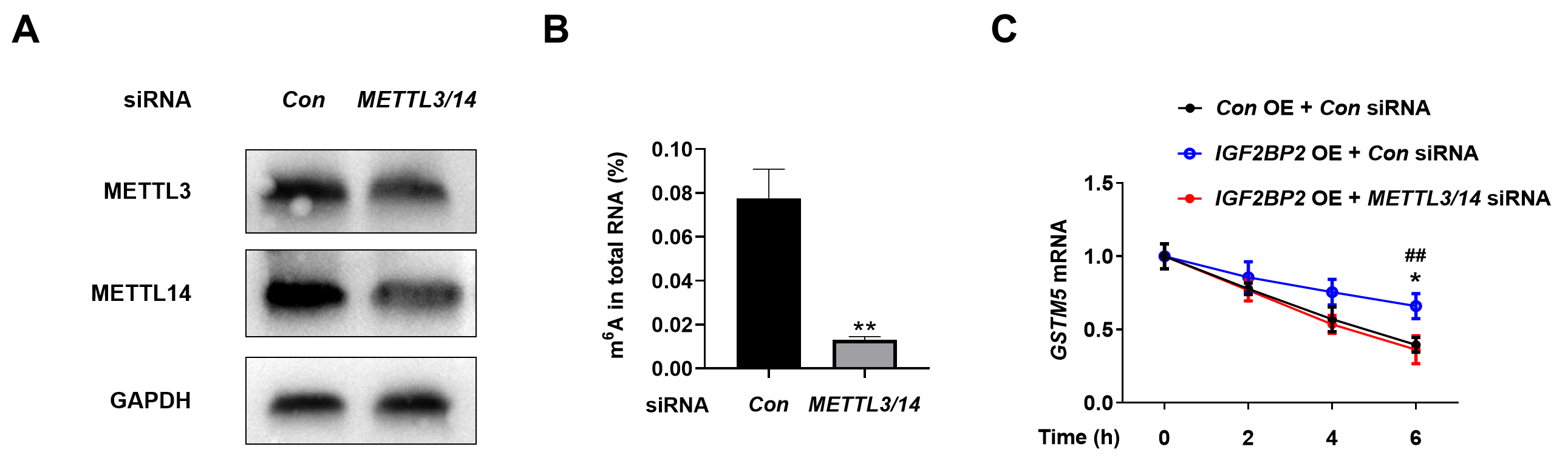


**Figure S13. M^6^A modification involved in IGF2BP2-mediated *GSTM5* mRNA stability. (A)** The expression of METTL3 and METTL14 in RA-FLSs with/without the *METTL3/14* siRNA transfection. **(B)** Total RNA m^6^A levels of RA-FLSs with/without the *METTL3/14* siRNA transfection. ^**^*P* < 0.01. **(C)** RA-FLSs were infected by *IGF2BP2* lentivirus and transfected by *METTL3/14* siRNA, and the mRNA expression of *GSTM5* was analyzed at the predetermined times following actinomycin D (5 μg/mL) treatment. ^*^Compared with *Con* OE + *Con* siRNA group. ^#^Compared with *IGF2BP2* OE + *METTL3/14* siRNA group. ^*^*P* < 0.05; ^##^*P* < 0.01.


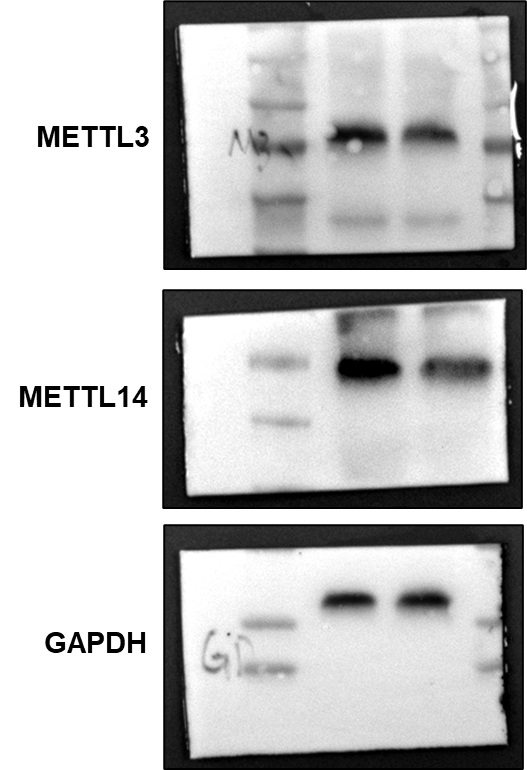


**Figure S14. Original blots in Figure S13.** Original blots in Figure S13A.


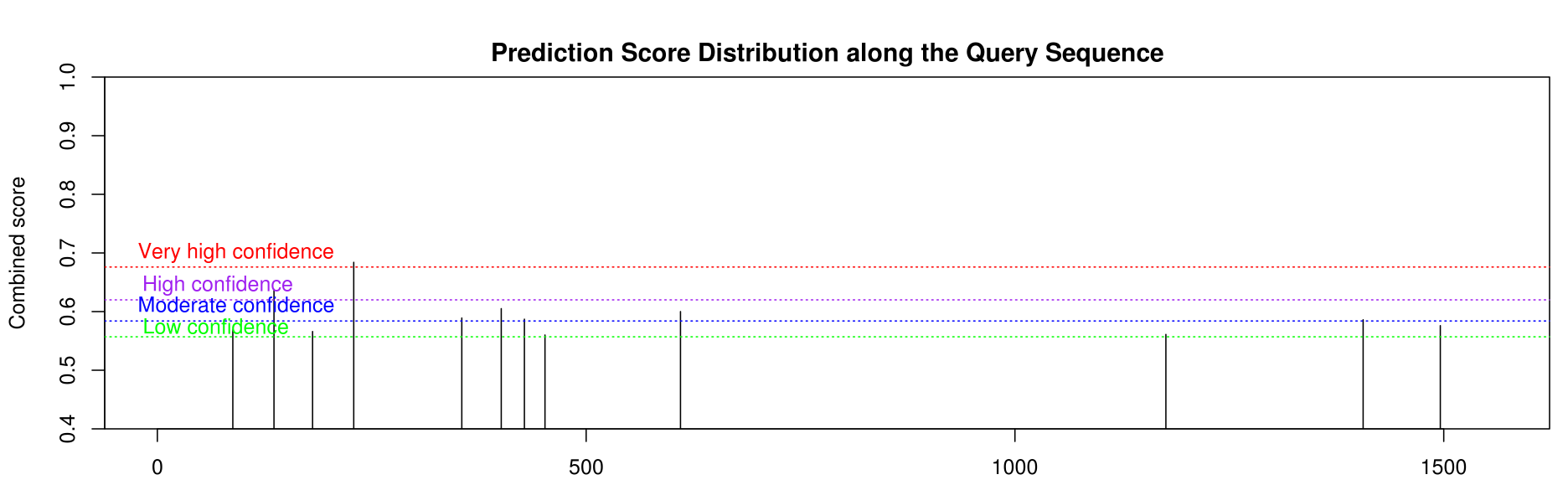


**Figure S15. Predictive m^6^A modification sites in *GSTM5* mRNA.** Prediction score distribution along the query sequence.


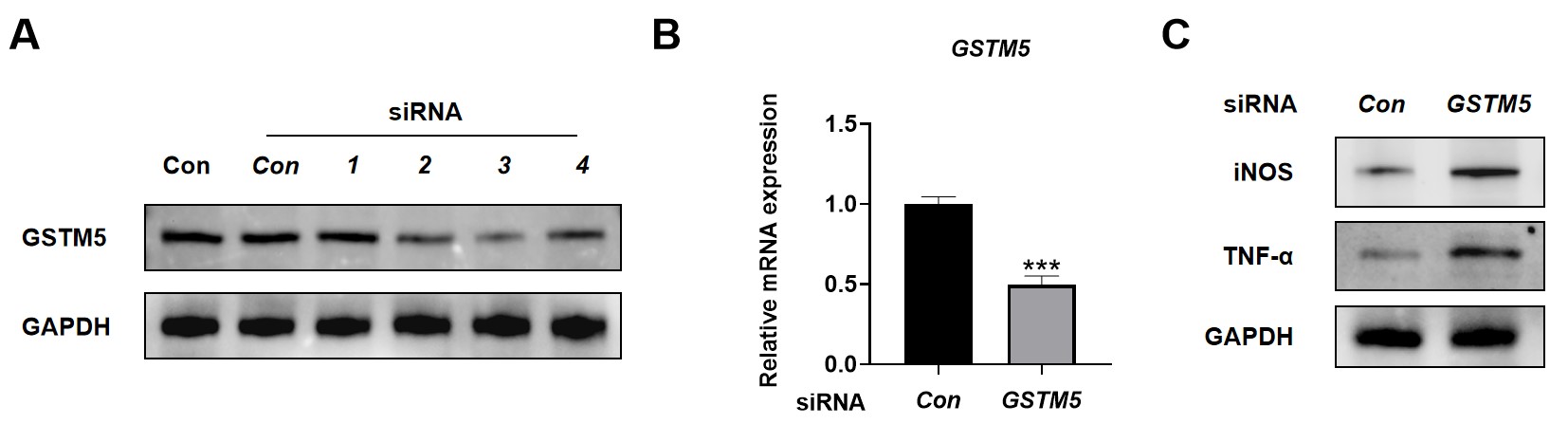


**Figure S16. The effect of *GSTM5* siRNA on the inflammatory response in RA-FLSs. (A)** Western blotting was used to detect GSTM5 protein level in RA-FLSs after treatment with *GSTM5* siRNAs. **(B)** The mRNA expression of *GSTM5* in RA-FLSs after treatment with *GSTM5* siRNA. **(C)** Western blotting was used to detect TNF-α and iNOS protein levels in RA-FLSs after treatment with *GSTM5* siRNA. ^***^*P* < 0.001.


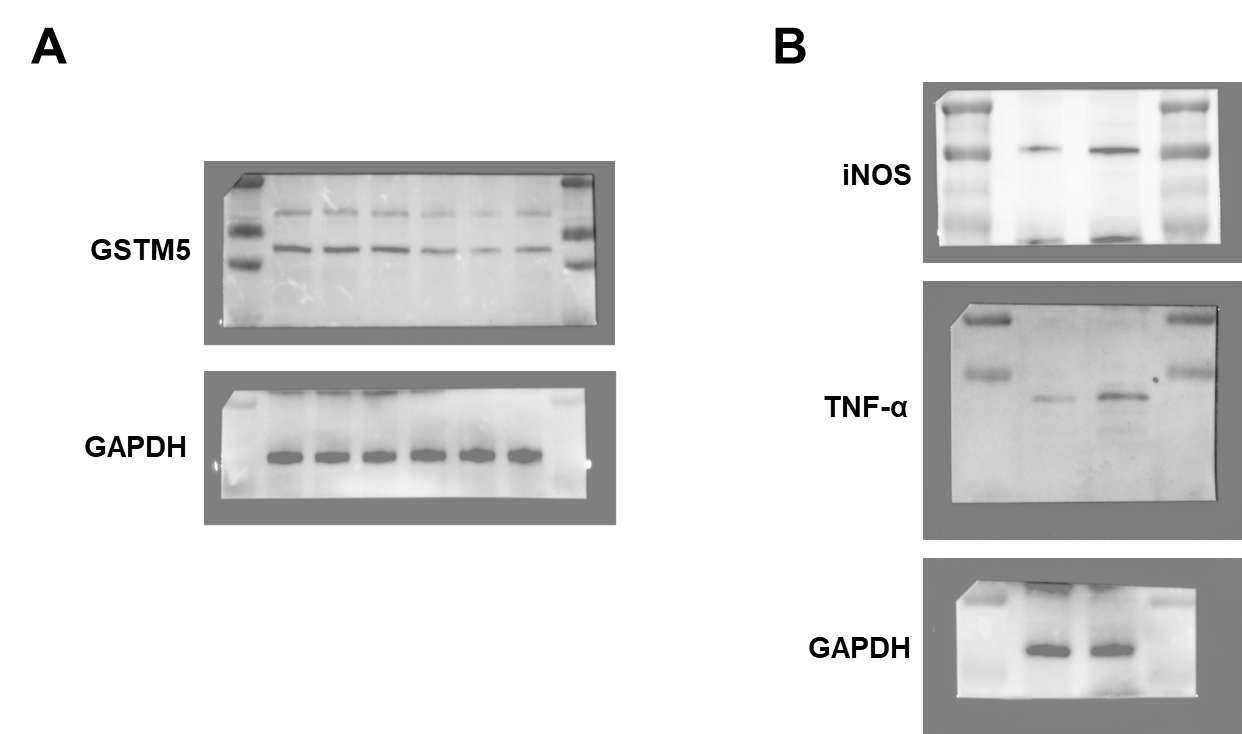


**Figure S17. Original blots in Figure S16. (A)** Original blots in Figure S16A. **(B)** Original blots in Figure S16C.


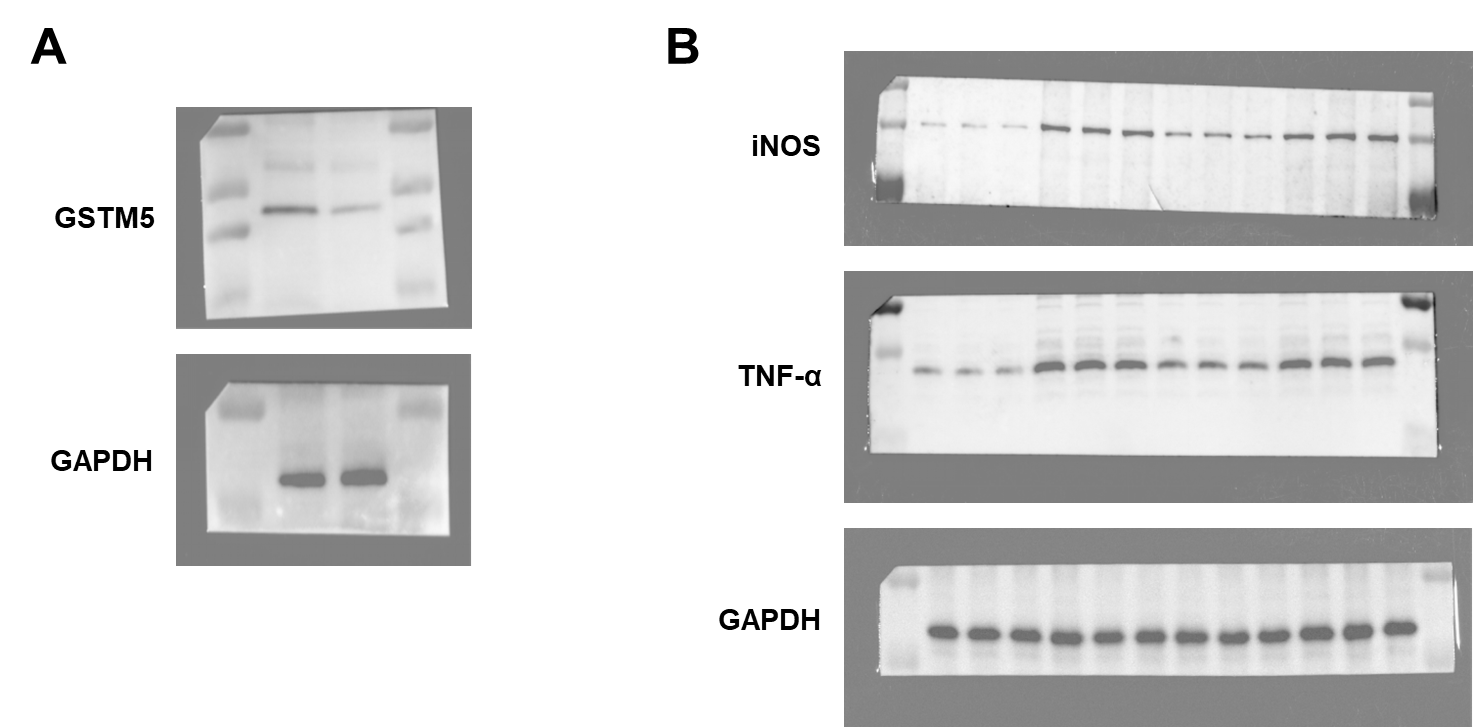


**Figure S18. Original blots in Figure 6.** (A) Original blots in Figure 6A. (B) Original blots in Figure 6L.


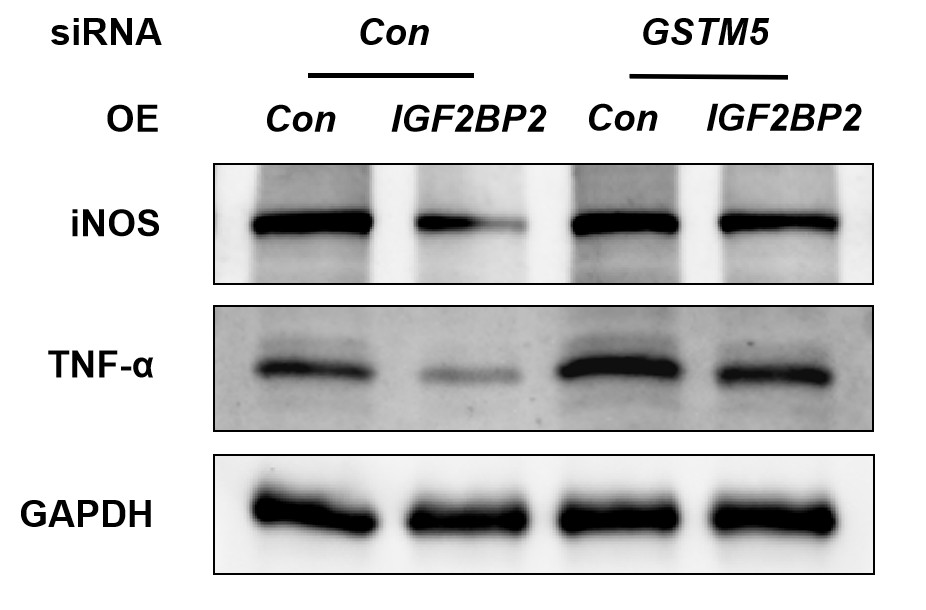


**Figure S19. The effect of IGF2BP2-GSTM5 axis on the inflammatory response in RA-FLSs.** Western blotting was used to detect TNF-α and iNOS protein levels in LPS-exposed RA-FLSs after treatment with *IGF2BP2* overexpressive lentivirus and *GSTM5* siRNA.


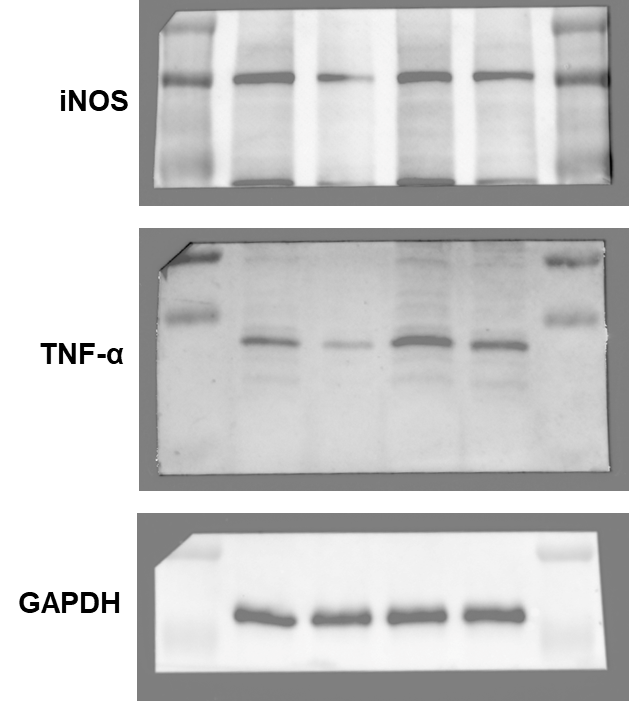


**Figure S20. Original blots in Figure S19.**

**
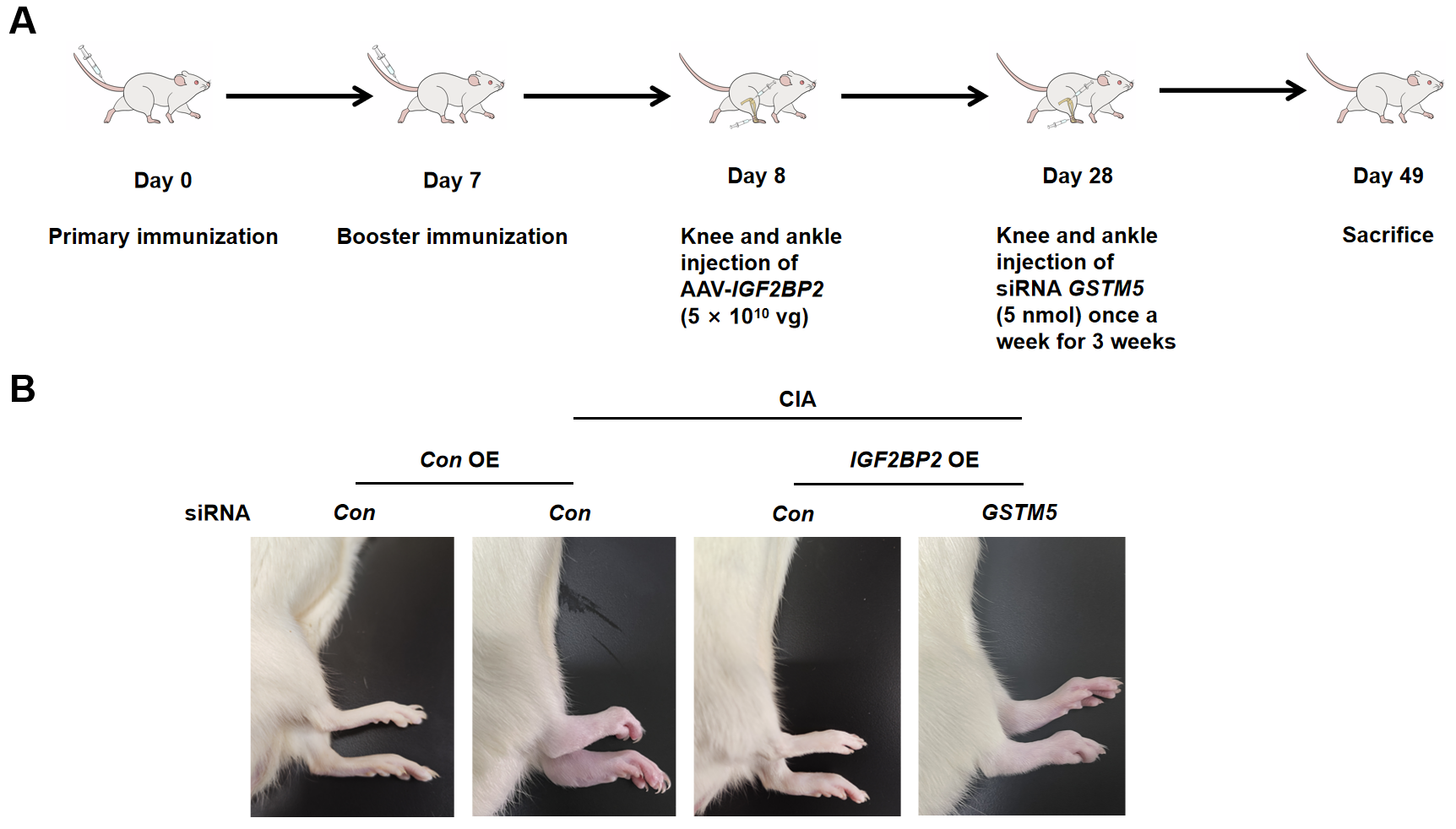
**

**Figure S21. The effect of IGF2BP2-GSTM5 axis on the severity of arthritis in CIA rats. (A)** Timeline of the animal experiment. **(B)** Representative photographs of morphology in different treatment groups.


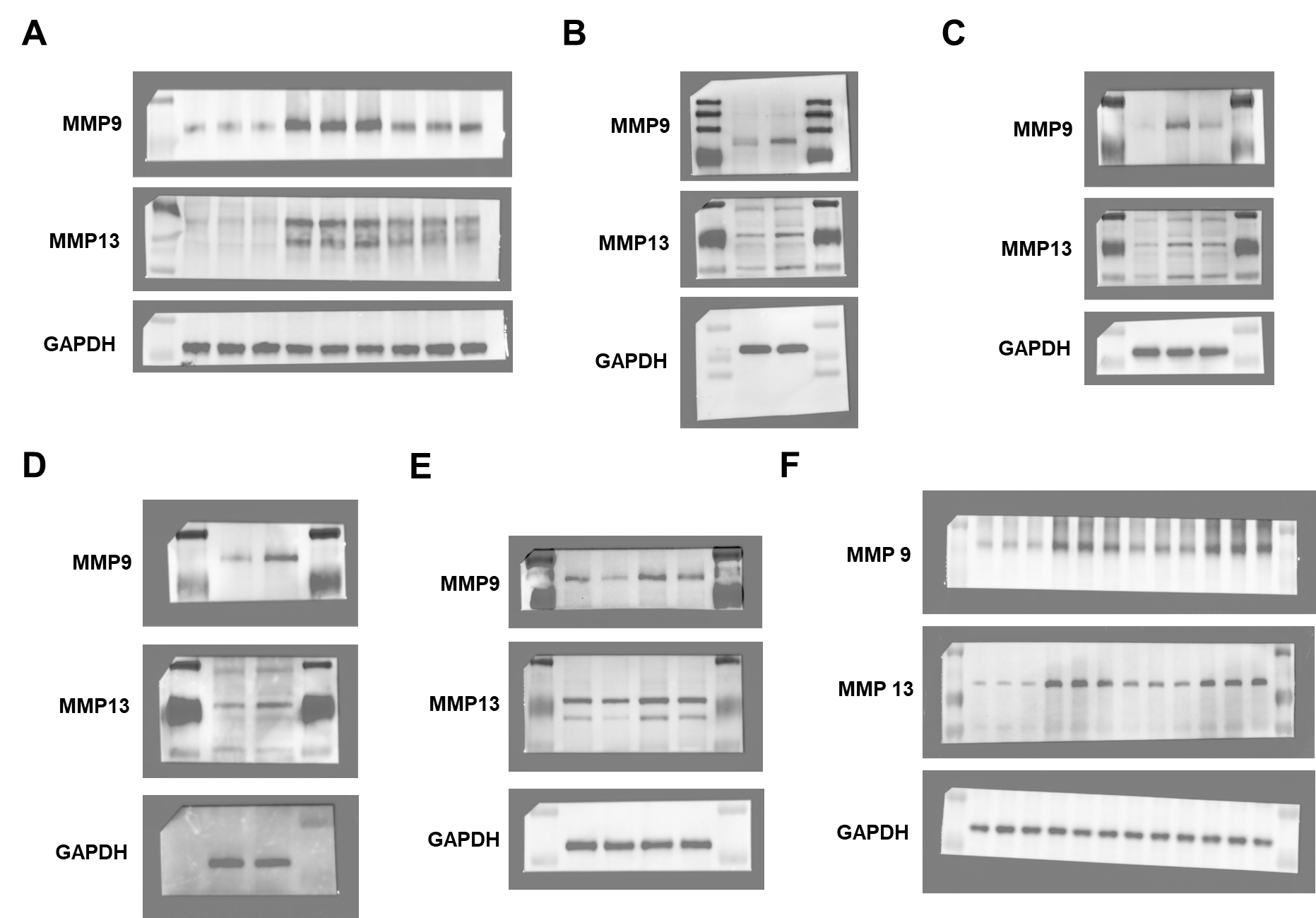


**Figure S22. Original blots in Figure 7. (A)** Original blots in Figure 7A. **(B)** Original blots in Figure 7B. **(C)** Original blots in Figure 7C. **(D)** Original blots in Figure 7D. **(E)** Original blots in Figure 7E. **(F)** Original blots in Figure 7F.
